# Enhanced Line Search Improves Robustness and Efficiency of Pose Sampling in Protein–Ligand Docking

**DOI:** 10.1021/acs.jctc.6c01110

**Published:** 2026-08-20

**Authors:** Leo Gaskin, Matthias Welsch, Johannes Kirchmair, Morteza Kimiaei

**Affiliations:** † Department of Pharmaceutical Sciences, 27258 University of Vienna, Vienna 1090, Austria; ‡ Christian Doppler Laboratory for Molecular Informatics in the Biosciences, Department of Pharmaceutical Sciences, University of Vienna, Vienna 1090, Austria; § Vienna Doctoral School of Pharmaceutical, Nutritional and Sport Sciences (PhaNuSpo), University of Vienna, Vienna 1090, Austria; ∥ Faculty of Mathematics, University of Vienna, Vienna 1090, Austria

## Abstract

Physics-based protein–ligand docking critically depends on efficient pose sampling, yet established sampling and local refinement algorithms can be inefficient and unstable in the highly nonconvex energy landscapes characteristic of protein–ligand interactions. To address this limitation, we introduce an enhanced local optimization strategy based on curved line search (CLS) and integrate it into AutoDock Vina, resulting in Vina_CLS. The proposed method enables more flexible step-size selection during local refinement and improves convergence in challenging regions of the energy landscape. Across benchmarks on the PDBbind refined set and the LEADS-PEP data set, Vina_CLS consistently outperforms the baseline, exhibiting greater robustness by solving more docking problems, as well as improved efficiency through reduced function and gradient evaluations and shorter runtimes. These gains translate into practical benefits, including more frequent identification of difficult-to-access local minima, enhanced redocking accuracy, and increased recovery of near-native poses. Together, these results demonstrate that improved local optimization can substantially enhance docking performance, highlighting an important, underexplored opportunity to advance structure-based drug discovery.

## Graphical abstract

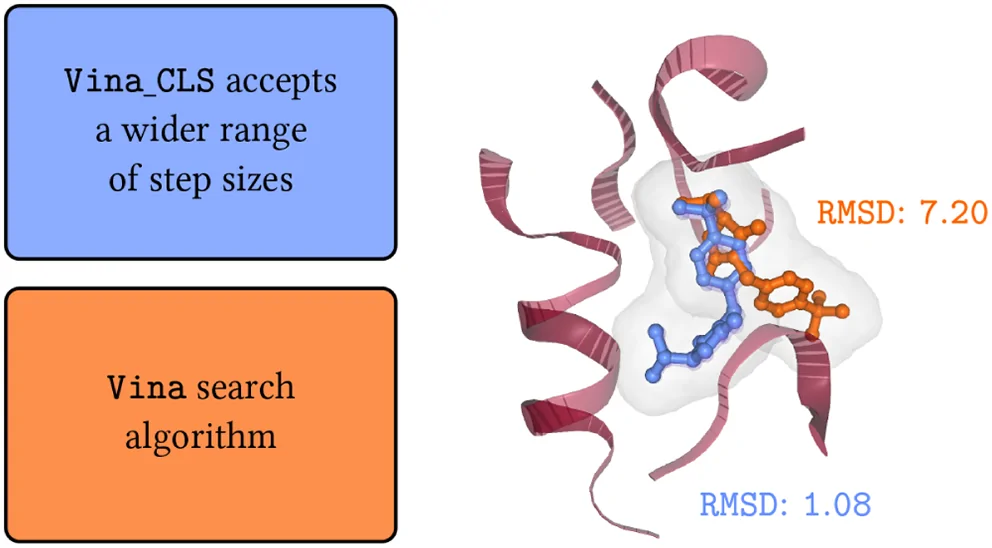

## 1 Introduction

Physics-based approaches for protein–ligand docking remain a mainstay in structure-based drug discovery, enabling the derivation of plausible ligand binding modes, virtual screening of large chemical libraries,^1^ and directed lead optimization.^2^ The docking process typically consists of two components: pose sampling and scoring. Pose sampling generates candidate ligand conformations within the protein binding site, exploring how the ligand may interact with the target. Scoring subsequently evaluates each proposed pose to estimate its compatibility with the binding site, typically based on energetic criteria.^3^

Finding the most likely ligand pose presents formidable optimization challenges, which have been addressed by a variety of approaches. Flexx^4^ was among the first docking programs, generating poses via discretization and a tree-search procedure that incrementally constructs ligand conformations through sequential fragment placement. While not relying on fragment-based assembly, Glide^5^ also employs discretization but instead performs exhaustive enumeration of conformations.

Pose sampling can also be carried out without discretization. Metaheuristic methods, such as genetic algorithms as used in GOLD,^6,7^ ant colony optimization techniques (e.g., PLANTS),^8,9^ and stochastic algorithms based on Monte Carlo simulations like AutoDock Vina^10,11^ directly explore continuous transformation spaces. In practice, pose sampling algorithms based on discretization or metaheuristic strategies are often complemented by local refinement techniques to improve candidate poses within promising regions of the search space.^8–11^

A major challenge in improving pose sampling algorithms arises from their tight coupling with scoring functions and their deep integration into the overall software.^12^ This challenge is further compounded by the prevalence of closed-source docking tools, which limit transparency and hinder modifications to the underlying methodologies.

The most widely used open-source docking tool,^13^ Vina, employs an iterative two-step procedure to identify favorable ligand poses. Each iteration begins with a Monte Carlo-based search to explore candidate conformations, followed by a BFGS-based local search that locally refines the pose. Consequently, at every Monte Carlo step, the proposed pose is tested for acceptance only after local optimization.

However, the Armijo line search^14^ exhibits notable limitations, particularly when applied to highly nonlinear energy landscapes such as those encountered in protein–ligand docking. In such settings, optimization algorithms may perform poorly, often selecting excessively large step sizes that cross energy barriers and lead to unstable convergence.^15,16^ Consequently, step-size heuristics have been adopted and developed in docking software. For example, variants of Vina^17,18^ include heuristics based on ref 19 to choose trial step lengths, and LSL-BFGS^15,16^ accepts a step only if it keeps per-atom displacements small.

To further mitigate excessive step size issues in line searches, additional descent conditions have been proposed to improve the robustness of pose sampling algorithms. For example, the RILC-BFGS algorithm introduced in Vina-GPU^20^ incorporates gradient information via the Wolfe condition^21^ to reduce overshooting during line search.

An alternative strategy to overcome the limitations of the Armijo line search is the curved line search (CLS).^22–24^ CLS employs a sufficient descent condition that is significantly less restrictive than the Wolfe or Armijo conditions, thereby accepting a broader range of meaningful step sizes. This property is particularly advantageous in strongly nonconvex regions, where it leads to more robust and efficient optimization performance (cf. ref 22–24 for details and numerical comparisons).

In this work, we present an enhanced line search algorithm derived from CLS and incorporate it into Vina, resulting in the software we refer to as Vina_CLS. The main scientific contributions are 3-fold: (1) we elucidate why accepting a broader range of step sizes is advantageous for protein–ligand docking, (2) we provide large-scale benchmark results showing that the proposed line search algorithm improves robustness by solving more problems and enhances efficiency by identifying better minima more rapidly, and (3) we show that the enhanced line search discovers more difficult-to-reach local minima and leads to improved redocking performance. To facilitate further research into pose sampling algorithms, we provide a Python interface that allows seamless integration of local search implementations into Vina.

## 2 Methods

Pose sampling in protein–ligand docking can be viewed as a nonlinear optimization problem (Figure 1). Considering a lower score indicating a better ligand pose, the goal of docking is to minimize a scoring function f_score_. A common design pattern of scoring functions is to decompose them into intermolecular (f_inter_) and intramolecular (f_intra_) contributions, defined with respect to a transformation **x** of the initial ligand conformation. This transformation includes a translation **t**, torsional angles **τ** around rotatable bonds, and a global rotation **q**, subject to the constraints that **t** lies within the bounding box [**t**_min_, **t**_max_], each of the *n*_τ_ torsional angle lies in [−π, π], and **q** is a valid rotation.

**Figure 1:**
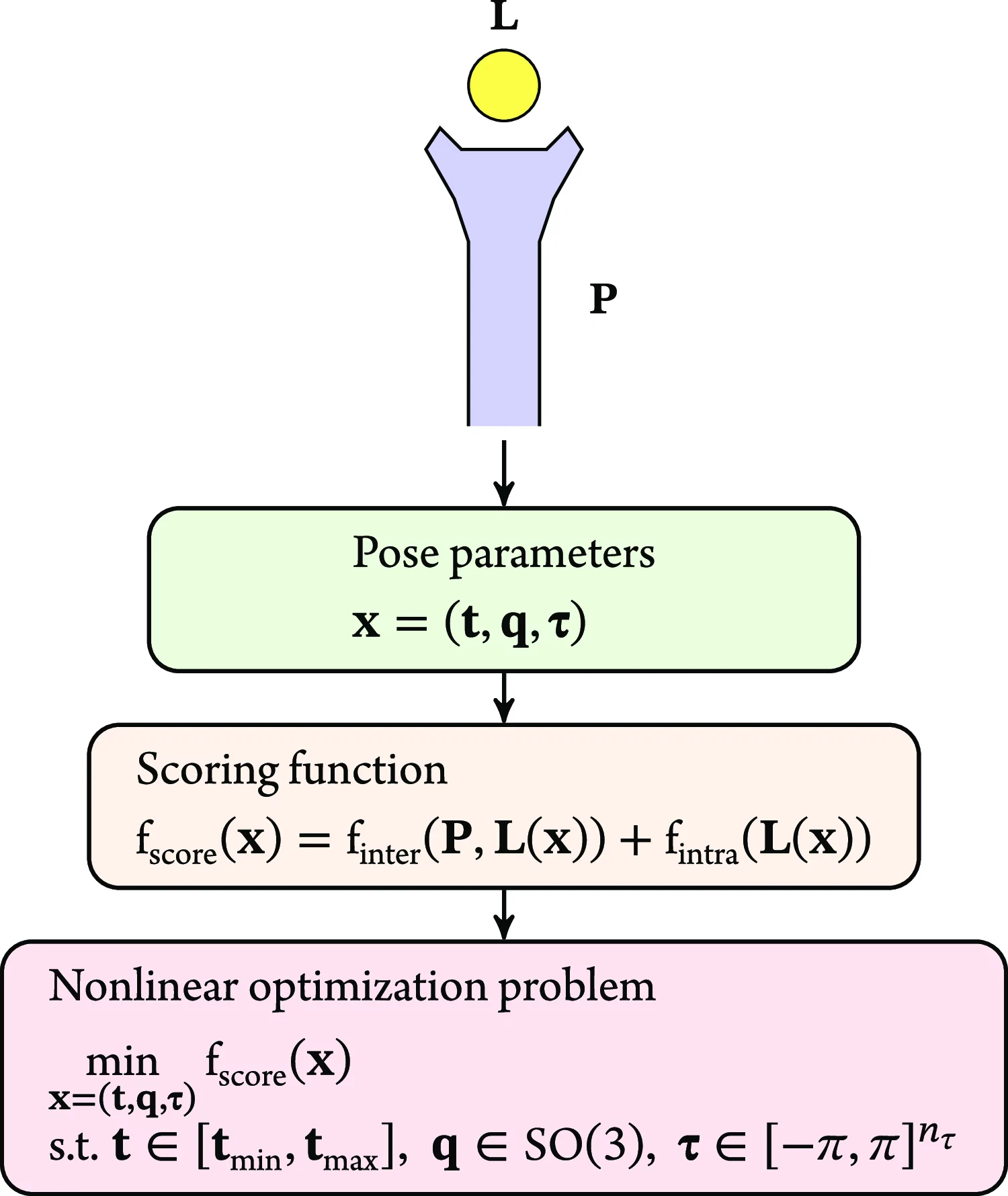
Conceptual representation of the protein–ligand docking problem as a nonlinear optimization task. The search space typically includes translational degrees of freedom and the rotational motion of the ligand (circle; **L**) relative to the binding site (custom horseshoe shape; **P**) on SO(3) as well as torsional angles within the ligand, making the resulting energy minimization problem highly nonconvex.

### 2.1 Pose Sampling with Vina: A Global–Local Search Scheme

To solve the nonlinear docking problem, Vina integrates a stochastic Monte Carlo global search with a deterministic local optimization (the Armijo line search performed along quasi-Newton directions) using the scoring function f_vina_. To efficiently explore the ligand-target energy landscape, a trial pose is generated by random perturbations in translation, rotation, or torsion. The pose is subsequently refined through local minimization using interpolation on precomputed grid maps based on continuous pairwise potentials.

The local refinement phase follows a standard BFGS update strategy^25^ and applies an Armijo-type backtracking line search.^14^ After local refinement, the resulting conformation is either accepted or rejected according to the Metropolis criterion before proceeding to the next iteration.

#### 2.1.1 The Armijo Line Search

Let **d** ≠ 0 be a descent direction, i.e., the descent condition

$$\nabla f{\left(x\right)}^{T}d<0$$ 1

holds. The step length α along the direction **d** is determined by an Armijo-type backtracking line search, which ensures a sufficient decrease in the objective function. Starting from an initial step size α_0_ > 0, the line search iteratively reduces α (typically by a factor β ∈ (0, 1)) until the Armijo condition

$$f\left(x+\alpha d\right)\leq f\left(x\right)+c\alpha \nabla f{\left(x\right)}^{T}d$$ 2

is satisfied, where *c* ∈ (0, 1) is a small constant. Equation 2 guarantees that each accepted step yields a sufficient decrease in energy. Performing Armijo-type line searches along BFGS directions provides a local optimizer, ensuring fast convergence while maintaining numerical stability.^21^

### 2.2 Improving the Local Search

A weakness of the Armijo line search is its conservative step-size selection (cf.^21^). This limitation becomes especially pronounced in docking, where the scoring function often features sharp potential walls that impede effective step-size selection.

The curved line search (CLS) method^23^ is an enhanced version of the Goldstein line search technique. Unlike the Wolfe line search, which uses gradient evaluations at multiple trial points, and like the Armijo line search, CLS uses only function values and the initial directional derivative to determine a sufficient descent step. Starting from an initial step size, the algorithm explores the search path **x**(α) ≔ **x** + α**d** along a descent direction **d** ≠ 0 and evaluates the Goldstein quotient

3 $$\mu \left(\alpha \right)≔\frac{f\left(x+\alpha d\right)-f\left(x\right)}{\alpha \nabla f{\left(x\right)}^{T}d}$$

Here, **d** is a descent direction, i.e., it satisfies the descent condition (eq 1). Intuitively, **d** forms an acute angle with the negative gradient, ensuring that small steps along **d** reduce the function value.

The goal of CLS is to find a step size α satisfying the sufficient descent condition (SDC)

μ(α)|μ(α)−1|≥β̅forafixedparameterβ̅∈(0,1/4) 4

Accordingly, $\mu \left(\alpha \right)=\frac{1}{2}$ is used as a natural target for two reasons. First, many smooth functions behave approximately like quadratic functions when close to a (local) minimum. In the quadratic case, one can verify that the optimal step size along a search direction satisfies $\mu \left(\alpha \right)=\frac{1}{2}$ . Thus, this value naturally represents a near-optimal choice in the regime where the algorithm is expected to converge (cf.^23^ Sec. 2.3, eq 22). Second, the left-hand side of the SDC (eq 4) is maximized when $\mu \left(\alpha \right)=\frac{1}{2}$ . This means that choosing μ close to $\frac{1}{2}$ leads to the strongest possible guarantee of progress in each step. Thus, μ­(α) can be interpreted as an indicator of step-size quality: values close to 1 correspond to overly small steps with slow progress, whereas values close to 0 correspond to overly large steps that may overshoot. The midpoint value $\frac{1}{2}$ , therefore, balances caution and progress.

Because $\frac{1}{2}$ is a natural target for the Goldstein quotient μ­(α) (eq 3), the search procedure aims to find α such that $\mu \left(\alpha \right)\approx \frac{1}{2}$ . Let [*α̲*, *α̅*] be a bracket with 0 < *α̲* < *α̅* < ∞. To obtain a step size α that yields a meaningful decrease in the objective function value, CLS performs an extrapolation step if $\mu \left(\alpha \right)\geq \frac{1}{2}$ (updating lower bound *α̲*); otherwise, if $\mu \left(\alpha \right)<\frac{1}{2}$ (updating upper bound *α̅*), it performs an interpolation step to enforce the SDC condition (in case the initial step satisfies the SDC, one additional interpolation is performed because μ­(α) is driven toward $\frac{1}{2}$ in the almost quadratic case, due to the guaranteed two-step termination on strictly convex quadratics^23^). In the worst case, when SDC cannot be met directly by either interpolation or extrapolation, CLS constructs [*α̲*, *α̅*] and iteratively refines it by updating either the lower or upper bound according to the function value at the new trial point. Once such a bracket is found, the next step size is chosen as the geometric mean of the bounds. If either extrapolation or interpolation yields a step size satisfying the SDC, the geometric–mean phase is skipped (Figure 2).

**Figure 2:**
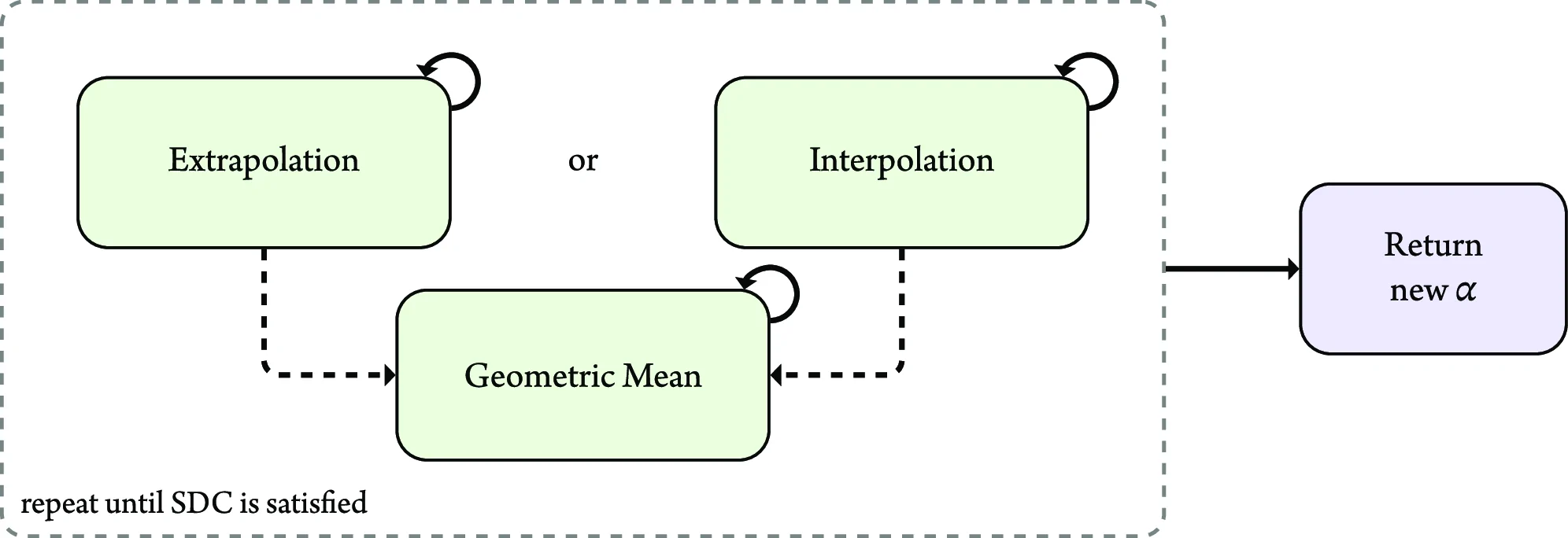
Conceptual workflow of the Curved Line Search (CLS) algorithm. The search iterates through either extrapolation or interpolation steps, followed by an optional geometric mean refinement. When the SDC is satisfied, or when a specified maximum number of iterations is reached, a step size α is returned.

The enhanced local search presented in this paper is based on the curved line search documented in Section S2 of the Supporting Information. For implementation details, see Section S3 of the Supporting Information. In Section 3.1, the choice of line search algorithm is motivated by discussing the implications of accepting a wide range of step sizes in protein–ligand docking applications.

### 2.3 Data Sets

This study validates Vina_CLS using the PDBbind^26^ refined set (1 starting position per problem; 5 random seeds) and LEADS-PEP^27^ (11 starting positions; 11 random seeds). The data sets were prepared using PDB2PQR^28^ for protein protonation and Meeko^29^ for converting proteins and ligands into the format required by Vina. Protein–ligand pairs that failed during any of these preprocessing steps (5 for LEADS-PEP and 468 for PDBbind) were excluded from the evaluation.

### 2.4 Evaluation Strategy

To assess the quality of Vina_CLS, we focus on the algorithm’s ability to generate a ligand pose with a low docking score. This strategy allows evaluating the effectiveness of the pose sampling process without conflating it with the quality of the scoring function. Although the docking score is only an approximation of the true energy landscape, it provides a consistent metric for measuring how effectively conformational space is explored to identify low-score poses.

Vina_CLS was evaluated for robustness (how many problems it can solve) and efficiency (how quickly it solves these problems compared to other solvers). To ensure a fair comparison with other solvers, our approach was evaluated according to community standards in the field of optimization.^30,31^

#### 2.4.1 Criteria for Declaring a Problem Solved

To determine whether a problem has been solved, it is insufficient to evaluate only the best point found, as the assessment also depends on the (unknown) global optimum. Typically, the quality of the initial guess is also considered and accordingly, performance is often measured using the ratio (*f*_*s*_ – *f*_tar_)/(*f*_0_ – *f*_tar_), which serves as an indicator of the convergence speed of the solver *s* in approaching a minimum of the smooth true function *f* (cf.^32^). Here, *f*_*s*_ denotes the best function value obtained by a solver *s*, while *f*_0_ is an initial guess of the function value shared by all solvers. Moreover, *f*_tar_ denotes the empirical target value used as a common reference for the problem, i.e., the best value attained on a problem across all numerical experiments (bona fide global minimum).

However, redocking experiments with Vina have two characteristics: (1) *f*_0_ is often uninformative due to protein–ligand clashes and (2) *f*_tar_ is usually in the very narrow range of −5 to −30 kcal/mol for **f**_vina_. For these reasons, we adopt

5 $$q_{s}≔f_{s}-f_{\mathrm{tar}}$$

from ref 33 as an interpretable rate of convergence specific to docking. For example, if $q_{s^{\prime }}=2$ for solver *s*′ with one seed, starting position, and budget on a given protein–ligand pair, then the best value found by that configuration is 2 kcal/mol higher than the best value achieved across all configurations for that pair.

A problem is considered solved by solver *s* if the rate of convergence *q*_*s*_ is less than or equal to the convergence threshold *ε*_*q*_ and a given budget β has not been exceeded. Otherwise, the problem is considered unsolved.

The budget is defined in terms of the maximum number of function evaluations nfmax, the maximum number of gradient evaluations ngmax, or the maximum allowed runtime timemax, in seconds.

#### 2.4.2 Relative Minimization Profiles as Robustness Indicators Under a Budget

β-relative minimization profile (β-RMP) plots^30^ are widely used in optimization to assess solver robustness. For a fixed budget β, a β-RMP depicts the proportion of problems solved as the convergence threshold varies. Presenting profiles for multiple budgets enables a direct comparison of methods in terms of both accuracy and computational cost.

#### 2.4.3 Performance Profiles as Measures of Solver Efficiency

To measure the efficiency for a fixed convergence threshold, the performance profile^31^ was employed. Let S denote the set of solvers under comparison and P the set of test problems, and let *c*_*p*,*s*_ be the cost measure (the number nf of function evaluations, the number ng of gradient evaluations, i.e., BFGS steps, and time in seconds time) of solver *s* for solving problem *p*. The performance profile of solver *s* measures the fraction of problems for which its performance ratio pr_*p*,*s*_ does not exceed the performance ratio threshold τ, i.e.,

6 $$\rho_{s}\left(\tau \right)≔\frac{1}{\left|P\right|}|\{p\in P\left|{\mathrm{pr}}_{p,s}≔\frac{c_{p,s}}{\min\left(c_{p,\mathrm{s̅}}|\mathrm{s̅}\in S\right)}\leq \tau \}\right|$$

In particular, ρ_*s*_(1) denotes the fraction of problems for which the solver *s* achieves the best performance among all solvers. For sufficiently large τ, ρ_*s*_ gives the fraction of problems that the solver *s* can solve, i.e., the robustness. By plotting ρ_*s*_(τ) against τ, performance profiles can illustrate an interpolation between the frequency at which a solver is most efficient and its robustness.

#### 2.4.4 Pose Sampling Comparison Details

There are two published local optimization methods available for protein–ligand docking with Vina. The first is the standard Vina optimizer, which employs a traditional line search algorithm with an Armijo-type condition.^11^ The second is the RILC-BFGS algorithm introduced as part of Vina-GPU.^20^ A comparison with the RILC-BFGS algorithm in Vina-CUDA was attempted, but differences in the scoring function implementation made a score-based comparison impossible (in some cases, for the same pose, the angle between the two implementations’ score gradients was greater than $\frac{\pi }{4}$ ). We have documented these differences in Section S4 of the Supporting Information. To fairly compare our implementation (Vina_CLS) with Vina, all pose samplers were initialized with the same random initial positions, and the random seeds were fixed. The exhaustiveness parameter was set to 1 to avoid ambiguity in counting function evaluations. The two algorithms were compared with β-RMP plots and by their performance profiles.

#### 2.4.5 Comparison with Cocrystallized Ligands

To compare which of two solvers (*s*_*a*_ and *s*_*b*_) can recover cocrystallized ligand geometry better for a given threshold *t*, the root-mean-square deviation (RMSD) to the native pose of all poses found by all solvers can be calculated across all problems (*s*(*p*) denotes all poses found for problem *p* with solver *s*). For each solver-problem combination, the minimum RMSD across poses is recorded. As a measure of how many additional problems *s*_*a*_ can solve compared to *s*_*b*_, define Δ­(*t*) as the difference in the number of problems with a minimum RMSD below *t* using the indicator function:

$$\Delta (t)=\sum_{p\in P}1\left[\underset{x\in s_{a}(p)}{\min}RMSD(x,p)<t\right]-1[\underset{x\in s_{b}(p)}{\min}RMSD(x,p)<t]$$ 7

A number larger than 0 indicates that *s*_*a*_ can recover the geometry of the cocrystallized ligands in the data set better than *s*_*b*_ for a given threshold *t*.

## 3 Results

This result section is structured in three parts. First, we illustrate, using a representative example, why accepting a broad range of step sizes is essential for protein–ligand docking. Second, we present large-scale benchmarks demonstrating that our approach achieves superior robustness and efficiency compared to the baseline. Third, we show that these improvements translate into better pose geometries: Vina_CLS identifies difficult-to-reach local minima and delivers improved redocking performance.

### 3.1 CLS Improves Step-Size Selection in Clash Regions

A previously encountered challenge in line searches, i.e., traversing transformation space along a given search direction (e.g., steepest descent) during protein–ligand docking concerns the handling of ligand poses in clash regions (i.e., regions where the geometries of ligand and protein overlap, resulting in infeasible regions).^15,16^ In such regions, gradients become very large, as even small conformational changes can produce substantial energy differences, leading to excessively large search directions.

The goal of a line search is to determine a scalar step size that scales the search direction such that the resulting point (i.e., ligand conformation) improves the objective (score). To avoid trivial updates (zero step size), a minimum permissible step size is typically enforced. However, when the search direction has very large components, even a minimum step size can produce substantial displacements, leading to drastic changes in ligand geometry.

Decreasing the minimum permissible step size is not always a viable option, as it risks trapping the algorithm in regions where the direction magnitude is small but nonzero. Therefore, a good line search for protein–ligand docking, where the gradient magnitude varies strongly, must be able to accept a wide range of step sizes to balance exploration in clash regions with exploitation in nonclash regions. Motivated by this observation, we base our enhanced line search on a curved line search approach specifically designed to provide this flexibility.

To illustrate the effect of step-size selection on ligand geometry, we contrast views of the energy landscape with the corresponding pose geometries. Specifically, we compare the paths taken by the line search utilized in Vina and our enhanced line search in Vina_CLS using a two-dimensional projection of the energy landscape around the pose geometries.

We generated the local optimizer paths by first perturbing the cocrystallized pose of imatinib in the C-ABL kinase domain (Figure 3, right column white ligand). From this perturbed starting position, we then employed both optimizers to minimize the score with a limited number of function evaluations.

**Figure 3:**
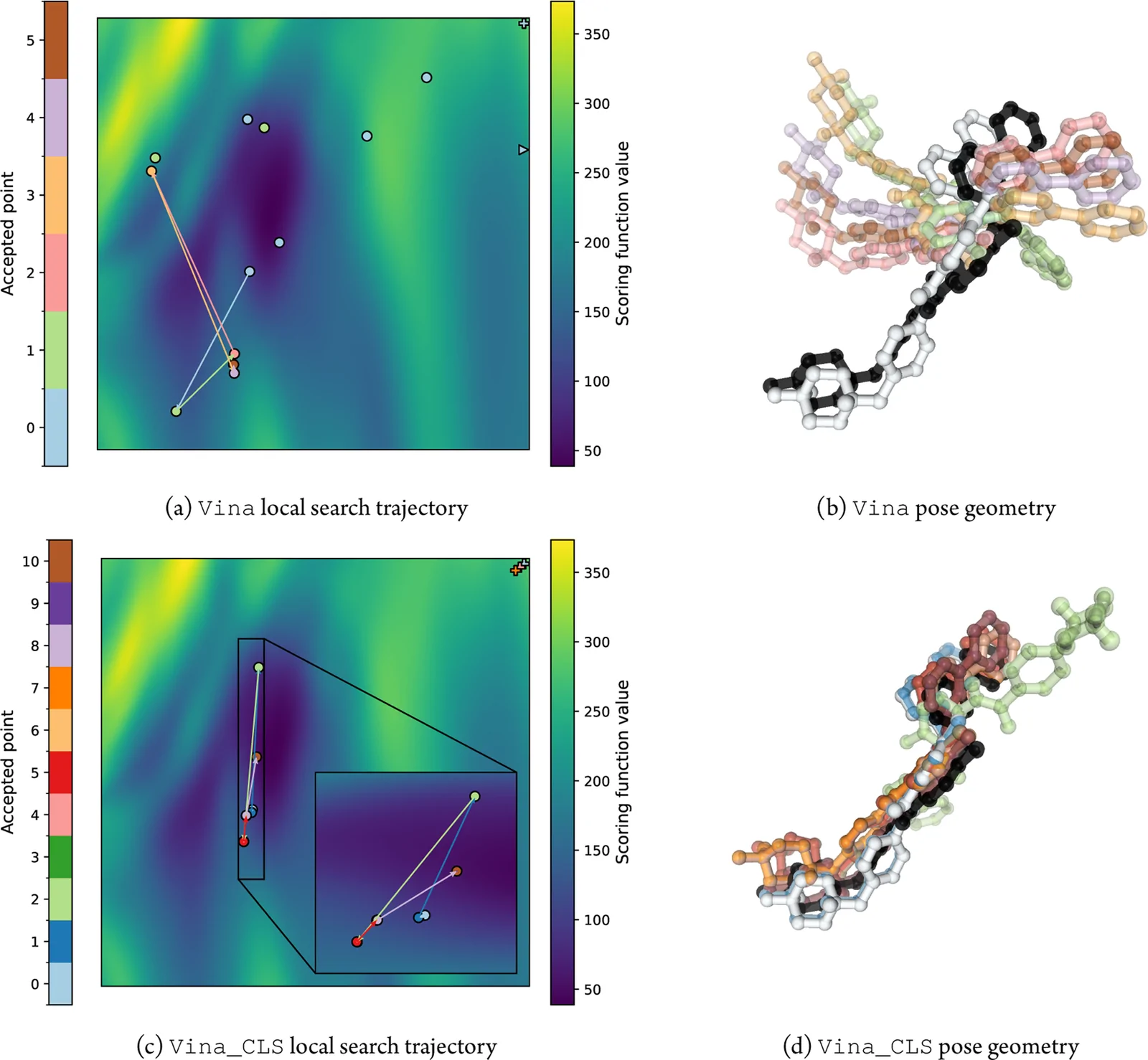
Impact of local optimization algorithm choice on optimization trajectory and pose geometry. In (a) and (c), the diagram’s background color indicates the scoring function value in the projected landscape, while the points indicate pose transformations evaluated during the optimization procedure. Initial points of successive BFGS iterations are connected by arrows, and their corresponding pose geometry is shown as a 3D molecular structure in (b) and (d) in the same color. Nonaccepted points share the color of the previous accepted point and are not connected by arrows. For clarity, the projected landscape has been cropped to the most relevant region with out-of-bounds points marked by noncircle symbols at the corresponding boundaries.

To obtain a visualization of the energy landscape, we first performed principal component analysis on the accumulated transformations (i.e., the extent to which the ligand has changed during optimization). We then sampled the space spanned by the two major principal axes uniformly to complement the scores obtained during optimization. Note that successive function evaluations during one line search are not on a line in this visualization, as large accumulated torsion angles and global rotation changes are themselves nonlinear.

Our chosen example illustrates a common failure mode of the local search algorithm in Vina that Vina_CLS is designed to avoid: Starting from a point with a high gradient, the line search algorithm of Vina failed to accept steps based on the descent condition and had to accept a degenerate step size (no function progress was made during all trial step sizes). However, given the large gradient, the transformation still escaped the local energy basin. In contrast, Vina_CLS was able to quickly accept a reasonable step, saving unnecessary function evaluations while remaining in the energy basin. Section S5 of the Supporting Information contains a more comprehensive quantitative evaluation of required steps until acceptance under realistic conditions.

### 3.2 Quantitative Evaluation of Solver Robustness and Efficiency

Having shown in one example that Vina_CLS exhibits favorable properties for step-size selection, we proceed to a more comprehensive evaluation. Specifically, to evaluate the performance of our implementation, we pursued a two-stage validation strategy. Similar to what is done in machine learning, we developed and tuned Vina_CLS on one data set and then validated it on a separate data set.

The LEADS-PEP^27^ data set was selected for developing the local search algorithm because it enables quick prototyping. Furthermore, peptides present a particularly challenging docking problem due to the large number of degrees of freedom (24 ± 12 rotatable bonds). To ensure the algorithm performs well in a realistic setting, we tested it on PDBbind, one of the most commonly used docking benchmarks (6 ± 5 rotatable bonds). Figure 4 shows a histogram comparing the number of rotatable bonds in both data sets, clearly showing the increased difficulty of the LEADS-PEP data set.

**Figure 4:**
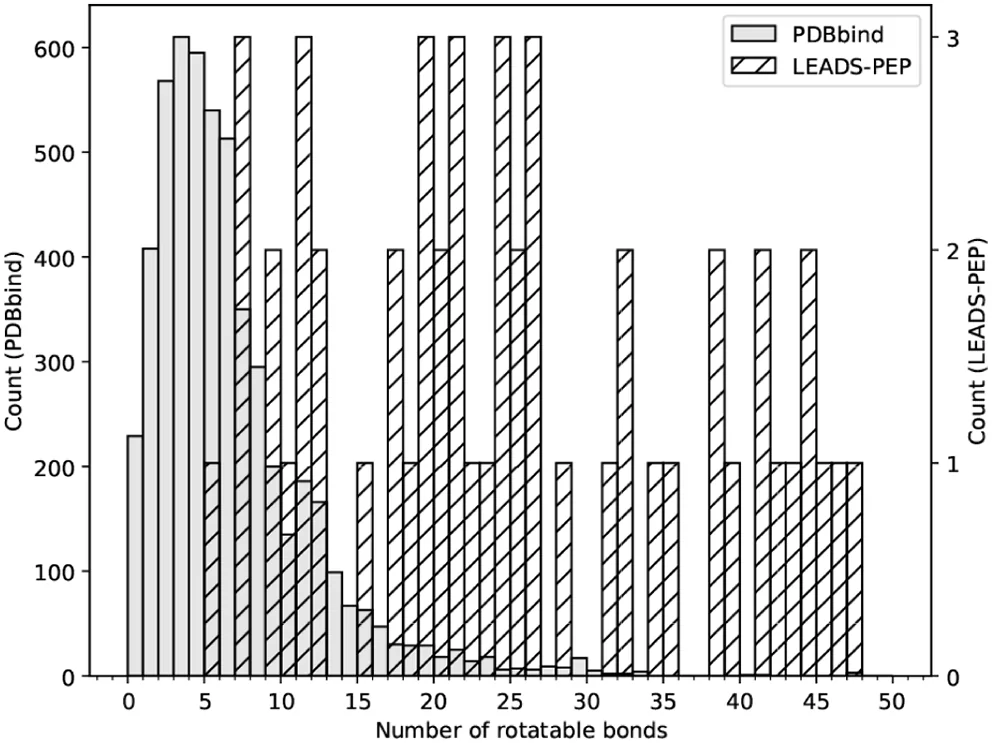
Number of rotatable bonds per ligand in PDBbind and LEADS-PEP.

To assess whether a problem is solved for a given convergence threshold, we calculated the difference from eq 5 using *f*_tar_ as the best value found across all numerical experiments for a protein–ligand pair. To assess robustness, we compute β-RMP plots (percentage of problems solved for a threshold; Section 2.4.1) for varying budgets β. Three budget sizes were explored: small, medium, and large. These were chosen so that a medium budget roughly corresponds to Vina’s (ligand-size dependent) default budget, with small being 10 times less and large being 10 times more. To assess efficiency, we considered performance profiles (interpolation between the frequency at which a solver is the most efficient and its robustness; eq 6).

#### 3.2.1 LEADS-PEP

We assessed the robustness and efficiency of Vina_CLS using the widely adopted LEADS-PEP docking benchmark data set. To account for stochasticity in the global Monte Carlo search, we considered 11 starting positions per protein–ligand pair, resulting in a total of 528 docking problems evaluated across 11 random seeds. Figure 5 shows a matrix of β-RMP plots for varying budgets β as a measure of robustness.

**Figure 5:**
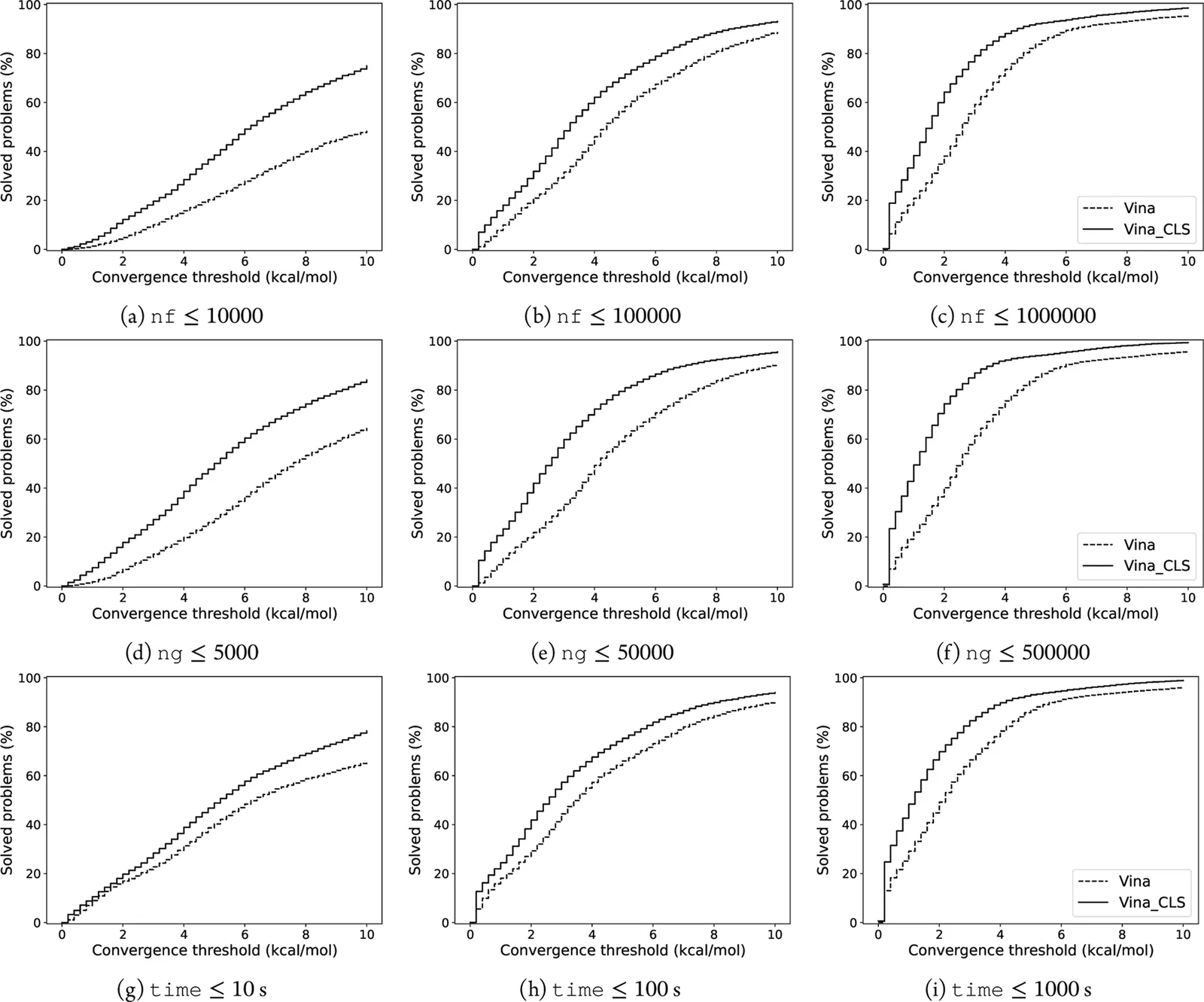
Robustness comparison of Vina and Vina_CLS on the LEADS-PEP benchmark using β-RMP across multiple budgets. Vina_CLS is more robust in most evaluated threshold regions and at least as robust in all threshold regions. Results are shown for increasing budgets of nf (a–c), ng (d–f) and time (g–i).

Notably, Vina_CLS was at least as robust as Vina across all evaluated budgets and thresholds, generally exhibiting a relevant increase in robustness. Except for small budgets, where a pronounced difference in robustness was observed even at nonstrict convergence thresholds, the difference in robustness increased as convergence thresholds became stricter. For example, with a threshold of 0.1 kcal/mol and a budget of 10^6^ function evaluations, Vina_CLS solved 15.12% of problems compared to 2.39% for Vina.

Having demonstrated that Vina_CLS performs more robustly in the optimization sense (most problems solved for various convergence thresholds), we turn to the question of the relevance of our improvement in terms of docking. We stipulate that a difference of ≈1 kcal/mol is still of moderate relevance to docking, as this error is typically tolerated in relative binding free energy calculations.^34^ Therefore, we defined 1 kcal/mol as the most relevant threshold and proceeded with our evaluation of efficiency at this threshold.

Given that Vina_CLS demonstrated greater robustness in the most relevant region (around a convergence threshold of 1 kcal/mol), we proceeded to evaluate the algorithm’s efficiency using performance profiles. Figure 6 shows the performance profiles of Vina and Vina_CLS (interpolation between the frequency at which a solver is the most efficient and its robustness at the highest budget) at a convergence threshold of 1 kcal/mol. We also paired problems by random seed, initial pose, and protein–ligand combination, and compared the cost of each solver with all paired solvers. If the convergence threshold was met by at least one paired solver, we call it paired solvable. We found Vina_CLS to be more efficient with respect to scoring function evaluations nf (in 83% of paired solvable cases) and gradient evaluations ng (in 90% of paired solvable cases). Notably, Vina_CLS was also more efficient with respect to time (in 76% of paired solvable cases), even though it is implemented via a computationally inefficient C++/Python bridge.

**Figure 6:**
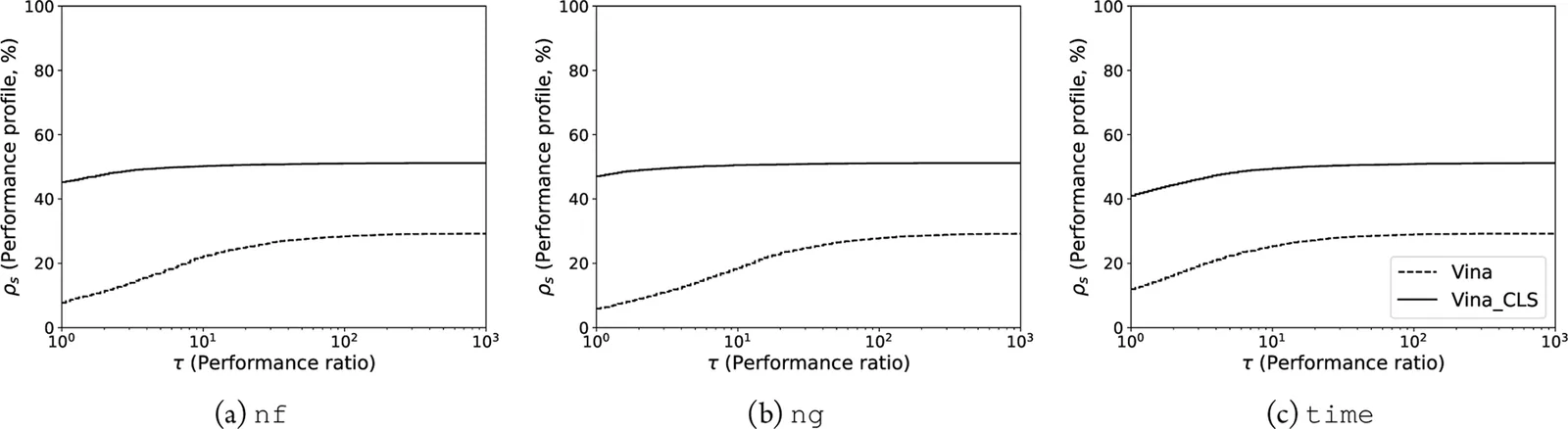
Performance profiles of Vina and Vina_CLS for LEADS-PEP benchmark based on the three cost measures nf (a), ng (b), and time (c) at a convergence threshold of 1 kcal/mol.

#### 3.2.2 PDBbind

Since we used LEADS-PEP during the development of Vina_CLS, we tested how well the proposed optimization procedure performs on new problems. Specifically, we investigated whether the algorithm performs well on the PDBbind refined set, the most commonly used docking benchmark data set. To account for algorithmic randomness, we ran all experiments with 5 random seeds.

Figure 7 reports the β-RMP plots for the PDBbind data set. It can be seen that the problems in PDBbind were easier to solve than those in LEADS-PEP for both algorithms. For example, at the highest nf budget (10^6^ function evaluations), Vina_CLS solved 89% of the problems to a convergence threshold of 1 kcal/mol, compared to only 38% for the LEADS-PEP benchmark. This is expected because of the decreased number of rotatable bonds, which reduces the degrees of freedom.

**Figure 7:**
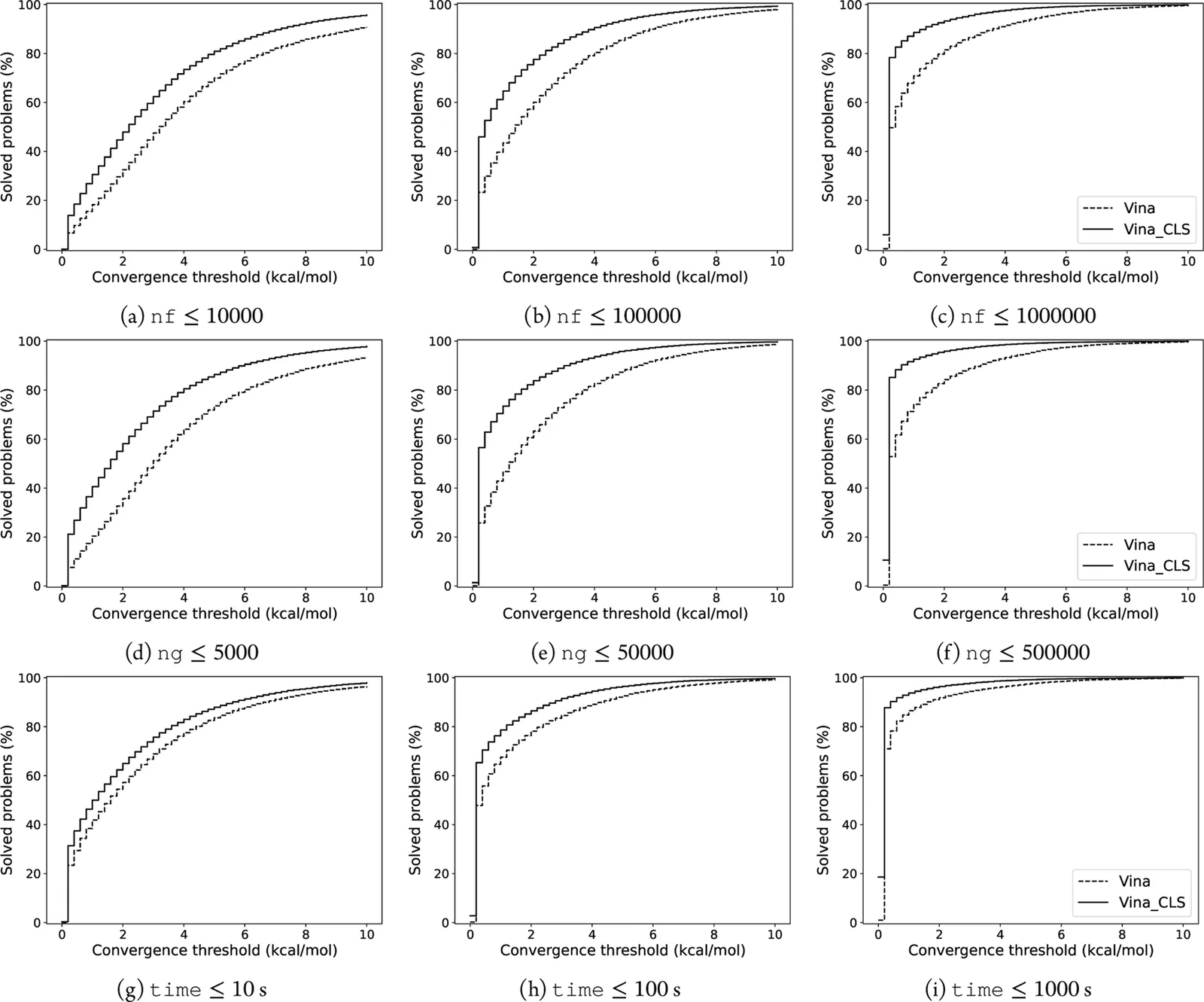
Robustness comparison of Vina and Vina_CLS on the PDBbind benchmark using β-RMP. Vina_CLS is more robust in most evaluated threshold regions and at least as robust in all threshold regions. Results are shown for increasing budgets of nf (a–c), ng (d–f) and time (g–i).

Like in the LEADS-PEP benchmark, Vina_CLS was generally more robust. In the PDBbind benchmark, we also observed greater improvement in robustness across medium budgets than in the LEADS-PEP benchmark, indicating that Vina_CLS can also enhance robustness for less challenging docking problems under restricted conditions.

We again performed our evaluation of docking efficiency at a convergence threshold of 1 kcal/mol. Also in this case, Vina_CLS was more efficient with regard to scoring function evaluations nf (in 73% of paired solvable cases), gradient evaluations ng (in 79% of paired solvable cases), and time (in 57% of paired solvable cases). However, the performance gain with respect to time was clearly smaller than for the LEADS-PEP benchmark, indicating that efficiency gains for the current implementation of Vina_CLS might be most pronounced for more challenging docking problems (Figure 8).

**Figure 8:**
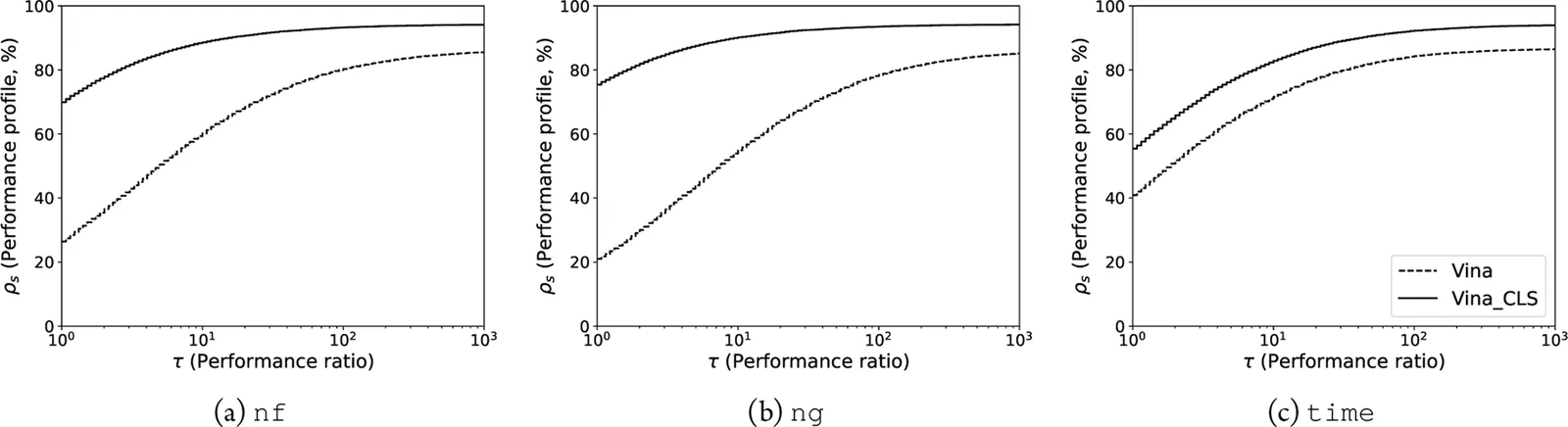
Performance profiles of Vina and Vina_CLS for PDBbind benchmark based on the three cost measures nf (a), ng (b), and time (c) at a convergence threshold of 1 kcal/mol.

### 3.3 Pose Evaluation

At this point, we have demonstrated that integrating the CLS algorithm improves robustness and efficiency of pose sampling in Vina. However, it remains unclear how these improvements translate to differences in pose geometry. To answer these questions, we performed an additional docking run consistent with a limited-budget application useful for virtual screening (8 initial positions, limit of 10^5^ nf scoring function evaluations) on the PDBbind refined set. For pose evaluation, we reuse the PDBbind data set prepared for our earlier experiments, i.e., 4850 prepared protein–ligand pairs.

#### 3.3.1 Vina_CLS May Find More Difficult-to-Reach Local Minima

To investigate how improvements in docking score translate to geometric differences between the poses found by each sampler, we asked whether Vina_CLS accesses geometrically distinct, lower-scoring local minima that are difficult to reach for Vina. We defined difficult-to-reach as geometric basins that only one solver found and to quantify geometrically distinct, we defined a threshold of 2 Å ligand RMSD; i.e., if the best pose obtained from Vina and the best pose obtained from Vina_CLS have a RMSD below 2 Å we consider them to occupy the same geometric basin.

To assess whether Vina_CLS found more lower-scoring, difficult-to-reach local minima, we partitioned the PDBbind ligands into two sets: 1026 ligands for which Vina achieved the lowest docking score, and 3824 ligands for which Vina_CLS did so. While 63% of pose pairs in the former set share a geometric basin by our definition, only 58% in the latter set do so. This suggests that when Vina_CLS attains the lowest score, it more often locates a different geometric basin, consistent with improved access to difficult-to-reach minima.

#### 3.3.2 Vina_CLS Exhibits Superior Docking Power Across Thresholds

Having established that Vina_CLS finds more geometrically distinct and lower-scoring local minima, we turn toward the question of whether these improvements lead to better performance in a more traditional docking sense (docking power). Specifically, we were interested in whether the poses found by Vina_CLS correspond better to the native poses of the cocrystallized ligand than the poses found by Vina. To this end, we defined Δ­(*t*), which counts how many more problems Vina_CLS solves within RMSD *t* compared to Vina (eq 7). We evaluated Δ­(*t*) for the top-1, top-3, and top-5 returned poses (those random seeds that achieved the lowest docking scores).

Across all experiments, Δ­(*t*) was larger than 0 for all thresholds *t* (Figure 9), indicating that Vina_CLS consistently recovered more poses within a RMSD threshold *t* than Vina in the top-1, top-3, and top-5 scenarios. At the conventional 2 Å cutoff this amounted to an improvement in recovery to 47% from 39% in the top-1 scenario, 50% from 44% in top-3, and 53% from 47% in top-5.

**Figure 9:**
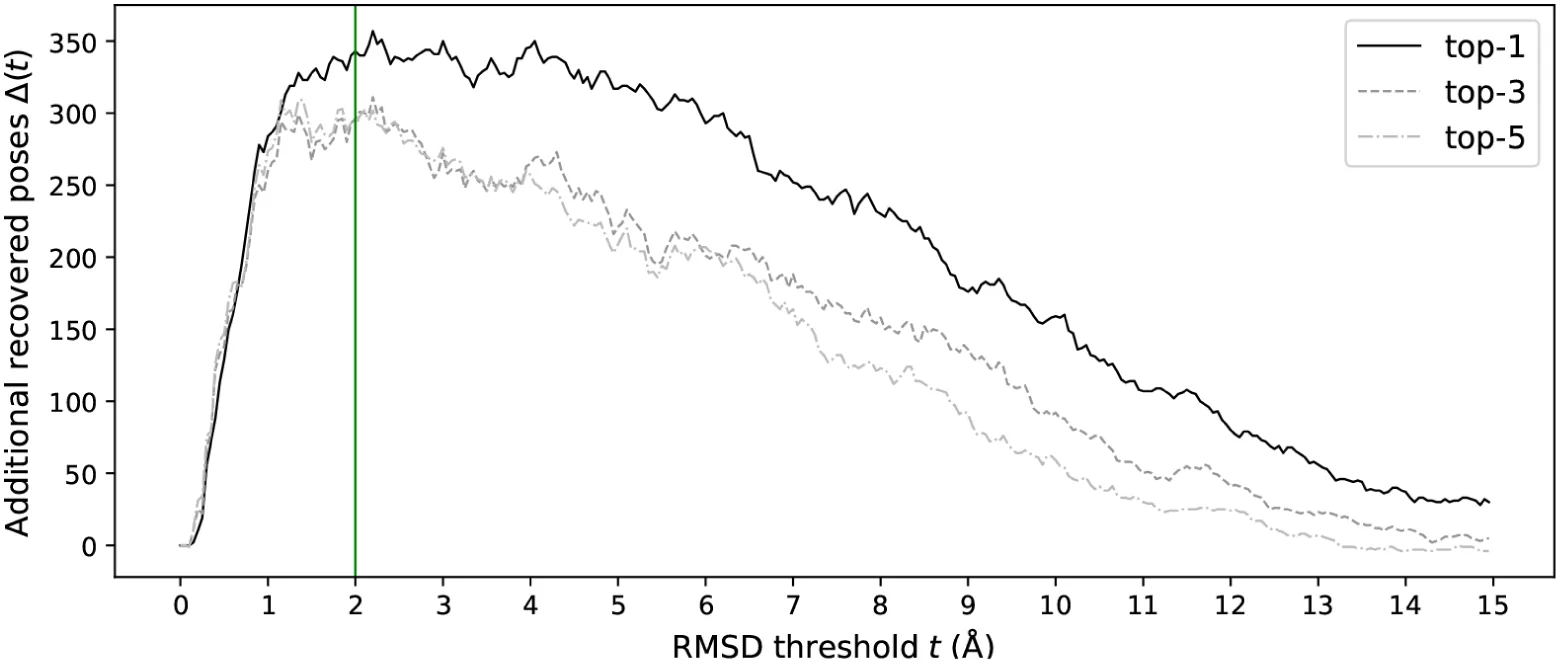
Additional poses found by Vina_CLS compared to Vina given various RMSD thresholds and top-k best scored poses. The conventional threshold of 2 Å is marked in green.

We provide an extensive stratified redocking analysis of the ligand-protein pairs based on ligand (number of rotatable bonds, number of heavy atoms) and protein (pocket size, pocket tightness) criteria in Section S6 of the Supporting Information. All attempted stratification strategies showed only a small impact on redocking performance, with a low number of rotatable bonds having the clearest impact. In particular, among ligands with fewer than three rotatable bonds, Vina_CLS shows an improvement in top-1 pose recovery Δ(2) relative to bin size of only 4% compared to 8% for all other ligands in the data set.

## 4 Conclusion

Building on CLS, we introduced an enhanced line-search strategy that substantially improves pose-sampling efficiency in one of the most widely used protein–ligand docking tools, Vina. The resulting method, Vina_CLS, demonstrated faster and more reliable convergence than the baseline Vina approach across two independent benchmark sets. Specifically, Vina_CLS achieved higher efficiency in up to 90% of paired solvable cases, depending on the evaluation criteria. Furthermore, pose quality improved, with an 8 percentage points increase in top-1 sub-2 Å pose recovery in our redocking benchmark. These results highlight the significant influence of the local optimization strategy on docking performance.

The improvements in algorithmic efficiency presented here not only facilitate the screening of larger compound libraries but also enable the reallocation of computational resources toward more accurate, albeit more computationally demanding, scoring functions.

To further support optimization-driven advances, we provide a Python interface that enables seamless integration of alternative local optimizers into Vina, thereby promoting broader experimentation at the intersection of numerical optimization and protein–ligand docking.

## Supplementary Material



## Data Availability

Data and Software Availability: This study used publicly available data from the PDBbind^26^ and LEADS-PEP.^27^ The source code of the approach presented in this work is available from https://github.com/leotaku/VinaCLS.

## Notes

Further details on the implementation of CLS, the Python interface to the local optimization algorithm, a comparison with Vina-CUDA, information about how many line search steps are required until acceptance within BFGS iterations, and a stratified analysis of redocking experiments (PDF)
