# Supplementary material for: Enhanced Line Search Improves Robustness and Efficiency of Pose Sampling in Protein–Ligand Docking
Source: J Chem Theory Comput. 2026 Aug 20;22(17):9188–98. doi: 10.1021/acs.jctc.6c01110 (PMC13564355; doi:10.1021/acs.jctc.6c01110)
Supplement: Supplementary file 1 [file ct6c01110_si_001.pdf]

Supporting Information for

Enhanced Line Search Improves Robustness and Efficiency of Pose Sampling in Protein–Ligand Docking

Leo Gaskin[a,†], **Matthias Welsch**[a,b,c,†], **Johannes Kirchmair**[a,b,⋆], **Morteza Kimiaei**[d,⋆]

[a] *Department for Pharmaceutical Sciences, University of Vienna, 1090 Vienna, Austria*
[b] *Christian Doppler Laboratory for Molecular Informatics in the Biosciences, Department for Pharmaceutical Sciences, University of Vienna, 1090 Vienna, Austria*
[c] *Vienna Doctoral School of Pharmaceutical, Nutritional and Sport Sciences (PhaNuSpo), University of Vienna, 1090 Vienna, Austria*
[d] *Faculty of Mathematics, University of Vienna, 1090 Vienna, Austria*

[†] Equal contribution
[⋆] Corresponding authors: johannes.kirchmair@univie.ac.at, morteza.kimiaei@univie.ac.at

Contents

S1 Introduction **S2**

S2 `CLS` **S2**

S2.1 Search Directions S2

S2.2 The `CLS` Algorithm S4

S3 Implementation details **S7**

S3.1 Required Adjustments in Finite Precision Arithmetic S7

S3.2 Python interface for custom local search algorithms in `Vina` S9

S4 Consistency of `Vina-CUDA` with `Vina` **S11**

S5 Distribution of line search steps until acceptance **S12**

S6 Stratified analysis of pose evaluation **S14**

References **S15**

S1 Introduction

This supplemental material provides detailed descriptions of the local optimization components used in our docking framework for the main paper. We present the search-direction formulations underlying the `BFGS`, `L-BFGS`, and `LM` methods; the complete specification of the `CLS` curved line search algorithm; and the numerical adjustments required to ensure robustness under finite-precision arithmetic. In addition, we document the implementation details needed to integrate custom local search procedures into `Vina` [1, 2], including the Python interface and data structures used for pose updates and tracing.

S2 `CLS`

This section discusses the three descent directions (`BFGS`, `L-BFGS`, and `LM`) and introduces pseudocode for `CLS`.

S2.1 Search Directions

The standard `BFGS` (`S-BFGS`) Direction. During the local phase, our advanced local optimization algorithm and `Vina` employ a quasi-Newton update scheme based on the Broyden–Fletcher–Goldfarb–Shanno (`BFGS`) method to generate search directions. Let $\mathbf{f}(\mathbf{x}) = \mathbf{f}_{\text{vina}}(\mathbf{x})$ denote the `AutoDock Vina` scoring function evaluated at the current ligand pose $\mathbf{x} = (\mathbf{t}, \mathbf{q}, \boldsymbol{\tau})$. At iteration k, the local minimizer maintains an approximation $\mathbf{B}_k$ to the Hessian matrix of second derivatives, from which the quasi-Newton search direction $\mathbf{d}_k$ is computed as

$$\mathbf{d}_k = -\mathbf{B}_k^{-1}\nabla\mathbf{f}(\mathbf{x_k}), \tag{1}$$

where $\nabla\mathbf{f}(\mathbf{x_k})$ is the exact gradient of f with respect to translation, rotation, and torsion variables. The matrix $\mathbf{B}_k$ is updated after each successful step according to the standard `BFGS` formula

$$\mathbf{B}_{k+1} = \mathbf{B}_k - \frac{\mathbf{B}_k\mathbf{s}_k\mathbf{s}_k^\top\mathbf{B}_k}{\mathbf{s}_k^\top\mathbf{B}_k\mathbf{s}_k} + \frac{\mathbf{y}_k\mathbf{y}_k^\top}{\mathbf{y}_k^\top\mathbf{s}_k},$$

where

$$\mathbf{s}_k := \mathbf{x_{k+1}} - \mathbf{x_k}, \quad \mathbf{y_k} := \nabla\mathbf{f}(\mathbf{x_{k+1}}) - \nabla\mathbf{f}(\mathbf{x_k}). \tag{2}$$

Since the approximate Hessian $\mathbf{B}_k$ is positive definite, the resulting $\mathbf{d}_k$ satisfies $\nabla\mathbf{f}(\mathbf{x_k})^\mathbf{T}\mathbf{d_k} < \mathbf{0}$, and therefore it is called a **descent direction**.

The `L-BFGS` Direction. The `L-BFGS` method is employed to determine an efficient quasi-Newton search direction without storing the full Hessian matrix. At iteration k, the `L-BFGS` direction $\mathbf{d}_k$ is computed as

$$\mathbf{d}_k = -\mathbf{H}_k\nabla\mathbf{f}(\mathbf{x_k}), \tag{3}$$

where $\mathbf{H}_k$ is an implicit approximation to the inverse Hessian, updated using a limited history of gradient and position differences. This approach provides a good trade-off between computational efficiency and curvature accuracy, making it suitable for large-scale optimization problems [3]. The L-BFGS method maintains a limited history of correction pairs $(\mathbf{s}_k, \mathbf{y}_k)$, where $\mathbf{s}_k$ and $\mathbf{y}_k$ are from (2). Using this information and defining $\rho_k := \dfrac{1}{\mathbf{y}_k^T \mathbf{s}_k}$, the inverse Hessian approximation is updated as

$$\mathbf{H}_{k+1} = \left(I - \rho_k \mathbf{s}_k \mathbf{y}_k^T\right) \mathbf{H}_k \left(I - \rho_k \mathbf{y}_k \mathbf{s}_k^T\right) + \rho_k \mathbf{s}_k \mathbf{s}_k^T.$$

The `LM` Direction. After each iteration, `LM` stores the correction pairs $(\mathbf{s}_k, \mathbf{y}_k)$ as the most recent columns of the matrices $\mathbf{S}_k := [\mathbf{s}_{k-m+1}, \ldots, \mathbf{s}_k]$ and $\mathbf{Y}_k := [\mathbf{y}_{k-m+1}, \ldots, \mathbf{y}_k]$. The symmetric matrix

$$\widetilde{\mathbf{H}}_k = \mathbf{S}_k^T \mathbf{Y}_k$$

is maintained to capture curvature information.

When the maximum memory size m is reached, the oldest columns of $\mathbf{S}_k$ and $\mathbf{Y}_k$ are deleted together with the corresponding row and column of $\widetilde{\mathbf{H}}_k$ to make room for the newest pair $(\mathbf{s}_k, \mathbf{y}_k)$. The augmentation

$$\mathbf{S}_k^+ = [\mathbf{S}_k\ \mathbf{s}_k], \qquad \mathbf{Y}_k^+ = [\mathbf{Y}_k\ \mathbf{y}_k],$$

produces the updated matrix

$$\widetilde{\mathbf{H}}_k^+ = \begin{pmatrix} \widetilde{\mathbf{H}}_k & \mathbf{h}_k \\ \mathbf{h}_k^T & \gamma_k \end{pmatrix}, \qquad \gamma_k = \mathbf{y}_k^T \mathbf{s}_k.$$

A Boolean variable `updateH` controls the computation of the coupling vector $\mathbf{h}_k$:

$$\mathbf{h}_k = \begin{cases} \mathbf{S}_k^T \mathbf{y}_k, & \text{if } \texttt{updateH} \text{ is true,} \\ \mathbf{Y}_k^T \mathbf{s}_k, & \text{otherwise.} \end{cases}$$

Even when the quasi-Newton condition $\mathbf{B}_k \mathbf{S}_k = \mathbf{Y}_k$ does not hold exactly, this update maintains symmetry of $\widetilde{\mathbf{H}}_k$ in finite-precision arithmetic.

The diagonal scaling matrix $\mathbf{D}_k \in \mathbb{R}^{n \times n}$ is estimated from the newest and oldest correction pairs as

$$(\mathbf{D}_{ii})_k := \sqrt{\frac{\sum_{j \in J} \texttt{YY}_{ij}}{\sum_{j \in J} \texttt{SS}_{ij}}},$$

where

$$\texttt{YY} := \mathbf{Y}_{IJ} \circ \mathbf{Y}_{IJ}, \qquad \texttt{SS} := \mathbf{S}_{IJ} \circ \mathbf{S}_{IJ},$$

J contains the indices of the newest and oldest pair $(\mathbf{s}, \mathbf{y})$, and $\circ$ denotes elementwise multiplication.

Using

$$\mathbf{U}_k := \mathbf{Y}_k - \mathbf{D}_k\mathbf{S}_k, \qquad \Sigma_k := \mathbf{U}_k^T\mathbf{S}_k,$$

the compact quasi-Newton approximation of the Hessian is formed as

$$\mathbf{B}_k := \mathbf{D}_k + \mathbf{U}_k\Sigma_k^{-1}\mathbf{U}_k^T.$$

The search direction $\mathbf{d}_k$ is defined as the solution of

$$\mathbf{B}_k\mathbf{d}_k = -\nabla\mathbf{f}(\mathbf{x_k}).$$

Direct computation of $\mathbf{B}_k^{-1}$ is expensive when n is large. To avoid this, the goal is to solve $\mathbf{B}_k\mathbf{d}_k = -\nabla\mathbf{f}(\mathbf{x_k})$ efficiently without explicitly forming or inverting $\mathbf{B}_k$. By defining

$$\mathbf{M}_k := \mathbf{U}_k^T\mathbf{D}_k^{-1}\mathbf{Y}_k = \Sigma_k + \mathbf{U}_k^T\mathbf{D}_k^{-1}\mathbf{U}_k,$$

and substituting the expression of $\mathbf{B}_k$ into the system above, the `LM` direction can be written as

$$\mathbf{d}_k = \mathbf{D}_k^{-1}(\mathbf{U}_k\mathbf{z}_k - \nabla\mathbf{f}(\mathbf{x_k})) \tag{4}$$

with $\mathbf{z}_k = \Sigma_k^{-1}\mathbf{U}_k^T\mathbf{D}_k^{-1}\nabla\mathbf{f}(\mathbf{x_k})$. Using $\mathbf{U}_k = \mathbf{Y}_k - \mathbf{D}_k\mathbf{S}_k$, the matrix $\mathbf{M}_k$ can be reformulated as

$$\mathbf{M}_k = \mathbf{U}_k^T\mathbf{D}_k^{-1}\mathbf{Y}_k = (\mathbf{Y}_k - \mathbf{D}_k\mathbf{S}_k)^T\mathbf{D}_k^{-1}\mathbf{Y}_k = \mathbf{Y}_k^T\mathbf{D}_k^{-1}\mathbf{Y}_k - \mathbf{S}_k^T\mathbf{Y}_k = \mathbf{Y}_k^T\mathbf{D}_k^{-1}\mathbf{Y}_k - \widetilde{\mathbf{H}}_k.$$

Here, $\widetilde{\mathbf{H}}_k = \mathbf{S}_k^T\mathbf{Y}_k$ is already available and, as discussed above, it is updated efficiently at each iteration to preserve symmetry in finite precision arithmetic. Since $\mathbf{M}_k$ is an $m \times m$ matrix with $m \ll n$, computing $\mathbf{z}_k$ from

$$\mathbf{z}_k = \mathbf{M}_k^{-1}\mathbf{U}_k^T\mathbf{D}_k^{-1}\nabla\mathbf{f}(\mathbf{x_k})$$

is inexpensive. This formulation preserves the symmetry and positive definiteness of $\mathbf{B}_k$ and ensures the descent property $\nabla\mathbf{f}(\mathbf{x_k})^\mathbf{T}\mathbf{d_k} < \mathbf{0}$, while avoiding the explicit inversion of the large matrix $\mathbf{B}_k$.

S2.2 The `CLS` Algorithm

Algorithm S1 [4] presents the `CLS` procedure, designed to determine a step size α satisfying the SDC

$$\mu(\alpha)|\mu(\alpha) - 1| \geq \beta \tag{5}$$

for a fixed parameter $\beta \in (0, 1/4)$. Unlike traditional line search methods, such as the **Goldstein** and **Wolfe** algorithms, which rely on multiple gradient evaluations or impose restrictive interval conditions, `CLS` operates using only function values and the initial directional derivative. `CLS` works as follows:

• The algorithm begins with initialization parameters α_{init} and $\alpha_{\max}$, bounded by the positive tuning parameters κ and λ to ensure a proper search interval, and with the directional derivative $\nu = -\nabla\mathbf{f}(\mathbf{x}(\mathbf{0}))^\mathbf{T}\mathbf{x}'(\mathbf{0}) > \mathbf{0}$ denoting the descent magnitude along the search path

$\mathbf{x}(\alpha)$.
• At each iteration, CLS computes the **Goldstein quotient** $\mu(\alpha) = (\mathbf{f}_0 - \mathbf{f}(\mathbf{x}(\alpha)))/(\alpha\nu)$ and evaluates whether the SDC is satisfied. If not, the algorithm dynamically adjusts the step size through one of three update strategies:
(i) **extrapolation** is done by increasing the step size as $\alpha \leftarrow \alpha Q$ if $\mu(\alpha) < 0.5$,
(ii) **interpolation** is done by updating $\alpha \leftarrow \frac{1}{2}\alpha/(1 - \mu(\alpha)))$ if $\mu(\alpha) > 0.5$, or
(iii) a **geometric mean** is done by computing $\alpha \leftarrow \sqrt{\underline{\alpha}\,\overline{\alpha}}$ once a valid bracket $[\underline{\alpha}, \overline{\alpha}]$ enclosing a feasible α is identified.

The Boolean variables `first` and `firstOk` are used to enforce the behavior described above. CLS2 initializes `first` = 1 and `firstOk` = 0. If the first iteration is efficient, `firstOk` is set to true. In the subsequent iterations, `first` is set to false. The Boolean variable `first` ensures that the initial iteration handles near-quadratic behavior optimally by testing whether the function approximates a convex quadratic form. The Boolean variable `firstOk` ensures that the line search terminates at the second iteration when the first iteration is efficient but the second iteration is inefficient; in this case, the values from the first iteration are restored. The process continues until the SDC is satisfied or the maximal step size $\alpha_{\max}$ is reached, after which the accepted step size α is returned.

By relying solely on function evaluations and a single gradient at the starting point, CLS significantly reduces computational cost while maintaining robust convergence properties. It provides a wider acceptance range of step sizes compared to Goldstein or Wolfe line searches, enabling faster progress and greater stability in nonconvex optimization landscapes. As demonstrated in [5], CLS achieves comparable or superior performance with fewer function evaluations, making it a reliable and efficient alternative for both derivative-based and derivative-free optimization frameworks

CLS does not terminate before completing the second step. Instead, it records the function value at the first trial point together with the associated step size. Hence, Algorithm S1 [4] is equivalent to the version used in [5, 6], except that, because it performs at least two function evaluations, it may accept the second trial step size rather than the first. Consequently, all properties established in [5] remain valid.

Algorithm S1 `CLS`, **curved line search**

Purpose: `CLS` finds a step size α with $\mu(\alpha)|\mu(\alpha)-1| \ge \beta$

Input: $\mathbf{d} = \mathbf{x}'(\mathbf{0})$ (search direction), $\mathbf{x}(\alpha) = \mathbf{x}(\mathbf{0}) + \alpha\mathbf{d}$ (search path), $\mathbf{f}_0 = \mathbf{f}(\mathbf{x}(\mathbf{0}))$ (initial function value), $\nu = -\nabla\mathbf{f}(\mathbf{x}(\mathbf{0}))^{\mathbf{T}}\mathbf{x}'(\mathbf{0})$ (minus directional derivative)

Tuning parameters: α_{init} (initial step size), $\alpha_{\max}$ (maximal step size), $\beta \in \,]0, \frac{1}{4}[$ (parameter for efficiency), $Q > 1$ (factor for extrapolation), $0 < \kappa < \lambda < \infty$ (parameters for choosing α_{init} and $\alpha_{\max}$).

Requirements: $\nu > 0$, $\frac{\kappa\nu}{\|\mathbf{d}\|^2} \le \alpha_{\text{init}} \le \alpha_{\max} \le \frac{\lambda\nu}{\|\mathbf{d}\|^2} < \infty$

Initialization: `first`=1; `firstok`=0, $\underline{\alpha} = 0$; $\overline{\alpha} = \infty$; $\alpha = \alpha_{\text{init}}$;

while 1 **do**
    ▶ compute $\mathbf{f}_a = \mathbf{f}(\mathbf{x}(\alpha))$ and the Goldstein quotient $\mu(\alpha) = (\mathbf{f}_0 - \mathbf{f}_a)/(\alpha\nu)$;
    if $\mu(\alpha)|\mu(\alpha)-1| \ge \beta$ **then** ▷ sufficient descent condition was satisfied
        if `first`, $\alpha_1 = \alpha$; $\mathbf{f}_1 = \mathbf{f}_a$; **else**, **break**; **end**
        `firstok` = `first`;
    end if
    if `firstok` and not `first`, $\alpha = \alpha_1$; $\mathbf{f}_a = \mathbf{f}_1$; **break**; **end**
    if $\mu(\alpha) > \frac{1}{2}$, ▶ $\underline{\alpha} = \alpha$;
    elseif $\alpha = \alpha_{\max}$, ▶ **break**;
    else, ▶ set $\overline{\alpha} = \alpha$; ▷ linear decrease or more
    end
    if `first`, ▷ initially check whether function is almost quadratic or not
        ▶ `first` = 0;
        if $\mu(\alpha) < 1$, ▶ $\alpha = \frac{1}{2}\alpha/(1-\mu(\alpha))$; **else** ▶ $\alpha = \alpha Q$; **end**
    else
        if $\overline{\alpha} = \infty$, ▶ expand to $\alpha = \alpha Q$; ▷ extrapolation was done
        elseif $\underline{\alpha} = 0$, ▶ compute $\alpha = \frac{1}{2}\alpha/(1-\mu(\alpha))$; ▷ interpolation was done
        else, ▶ calculate $\alpha = \sqrt{\underline{\alpha}\,\overline{\alpha}}$; ▷ geometric mean was computed
        end
    end
    ▶ restrict $\alpha = \min(\alpha, \alpha_{\max})$;
    end
end while
return α and $\mathbf{f}_a$;

S3 Implementation details

In this section, we describe the implementation aspects necessary for incorporating custom local search procedures into `Vina`, with particular emphasis on the Python interface and the data structures supporting pose updates and tracing.

S3.1 Required Adjustments in Finite Precision Arithmetic

Let $\mathbf{x_k} \in \mathbb{R}^\mathbf{n}$ be the current point, $\mathbf{f}_k = \mathbf{f}(\mathbf{x_k})$ the current function value, and $\nabla \mathbf{f}(\mathbf{x_k})$ the current gradient. In what follows, we introduce several adjustments to the search directions and to `CLS` that are necessary to ensure robustness under finite-precision arithmetic.

Adjustments on Search Directions. To ensure that the descent condition

$$\nabla \mathbf{f}(\mathbf{x_k})^\top \mathbf{d_k} < \mathbf{0}$$

at $\mathbf{x_k}$ holds under finite-precision arithmetic, the following safeguards are applied. We denote by $[\mathbf{v}]_i$ the ith component of the vector $\mathbf{v}$. If

$$\nabla \mathbf{f}(\mathbf{x_k})^\top \mathbf{d_k} \geq \mathbf{0} \quad \text{and} \quad \nabla \mathbf{f}(\mathbf{x_k})^\top \mathbf{d_k} \text{ is not } \texttt{NaN},$$

then we define the index set

$$\mathcal{I} = \{\, i \mid [\nabla \mathbf{f}(\mathbf{x_k})]_\mathbf{i} [\mathbf{d_k}]_\mathbf{i} > \mathbf{0} \,\}$$

and distinguish two cases:
Case a. If $\mathcal{I} \neq \emptyset$, we flip the corresponding components of the search direction:

$$[\mathbf{d}_k]_i \leftarrow -[\mathbf{d}_k]_i, \quad \forall i \in \mathcal{I}, \qquad \nabla \mathbf{f}(\mathbf{x_k})^\top \mathbf{d_k} \leftarrow -\, \nabla \mathbf{f}(\mathbf{x_k})^\top \mathbf{d_k}.$$

Case b. If $\mathcal{I} = \emptyset$, use the steepest descent direction:

$$\mathbf{d}_k \leftarrow -\nabla \mathbf{f}(\mathbf{x_k}), \qquad \nabla \mathbf{f}(\mathbf{x_k})^\top \mathbf{d_k} = -\|\nabla \mathbf{f}(\mathbf{x_k})\|^\mathbf{2}.$$

If, after these two cases, we check whether still

$$\nabla \mathbf{f}(\mathbf{x_k})^\top \mathbf{d_k} \geq \mathbf{0} \quad \text{or} \quad |\nabla \mathbf{f}(\mathbf{x_k})^\top \mathbf{d_k}| \approx \mathbf{0}.$$

If yes, we apply a small perturbation to guarantee a strict descent direction:

$$\mathbf{d}_k \leftarrow -\, \nabla \mathbf{f}(\mathbf{x_k}) - \mathbf{10^{-8}} \operatorname{sign}(\nabla \mathbf{f}(\mathbf{x_k})).$$

On the other hand, if $\nabla \mathbf{f}(\mathbf{x_k})^\top \mathbf{d_k}$ is `NaN`, we reset the direction:

$$\mathbf{d}_k \leftarrow -\nabla \mathbf{f}(\mathbf{x_k}), \qquad \nabla \mathbf{f}(\mathbf{x_k})^\top \mathbf{d_k} = -\|\nabla \mathbf{f}(\mathbf{x_k})\|^\mathbf{2}.$$

These adjustments ensure that, even under finite-precision arithmetic, the computed search direction $\mathbf{d}_k$ always satisfies the descent condition $\nabla \mathbf{f}(\mathbf{x_k})^\top \mathbf{d_k} < \mathbf{0}$.

When the search direction $\mathbf{d}_k$ is obtained by solving

$$\mathbf{B}_k \mathbf{d}_k = -\nabla \mathbf{f}(\mathbf{x_k}),$$

finite-precision arithmetic may cause numerical instability if the approximate Hessian $\mathbf{B}_k$ is ill-conditioned or nearly singular. To ensure numerical stability and preserve the descent property, a small regularization term is added to the diagonal of $\mathbf{B}_k$, yielding the modified system

$$\Big(\mathbf{B}_k + \varepsilon \mathbf{I}\Big)\mathbf{d}_k = -\nabla \mathbf{f}(\mathbf{x_k}), \qquad \varepsilon = \mathbf{10^{-4}}.$$

The addition of $\varepsilon\mathbf{I}$ guarantees that the matrix $\mathbf{B}_k + \varepsilon\mathbf{I}$ is positive definite and well-conditioned, thereby avoiding numerical breakdowns and ensuring that a stable and consistent search direction $\mathbf{d}_k$ is computed even under finite-precision arithmetic.

Estimation of the predicted differences $\Delta\mathbf{f}_k$ of function values. At initialization, the value of $\Delta\mathbf{f}_k$ is defined by

$$\Delta\mathbf{f}_0 = \begin{cases} \mathrm{fac}_f * |\mathbf{f}_0|, & \text{if } \mathbf{f}_k \text{ is finite and } \mathbf{f}_0 \neq 0, \\ 1, & \text{otherwise,} \end{cases}$$

where $\mathrm{fac}_f > 0$ is a tuning parameter. During the iteration, let $\mathbf{f}_k$ denote the current function value, $\mathbf{f}_{k-1}$ the previous function value, and $\mathbf{f}_{\mathrm{best}}$ the best function value found so far. Defining the indicator

$$\mathrm{dec} = \begin{cases} 1, & \text{if } \mathbf{f}_k < \mathbf{f}_{k-1}, \\ 0, & \text{otherwise,} \end{cases}$$

$\Delta\mathbf{f}_k$ is updated according to

$$\Delta\mathbf{f}_k = \begin{cases} \mathbf{f}_{k-1} - \mathbf{f}_k, & \text{if dec} = 1, \\ \min\Big(\max\Big(\max\{\, \Delta_f\, \Delta\mathbf{f}_{k-1},\ \Delta_m\,(|\mathbf{f}_{k-1}| + |\mathbf{f}_k|)\,\},\ 10^{-8}\Big),\ 10^{8}\Big), & \text{if dec} = 0, \end{cases}$$

where $\Delta_f > 0$ and $\Delta_m > 0$ are the tuning parameters, and the bounds 10^{-8} and 10^{8} ensure numerical stability and prevent overflow. If any intermediate quantity is non-finite, it is replaced by a large constant value. $\Delta\mathbf{f}_k$ is used in the adjustment of `CLS` in finite precision arithmetic, below.

Adjustments on `CLS` as in [6] When, due to finite-precision arithmetic, the SDC condition (5) cannot be satisfied, even though it is theoretically valid (cf. [5]), the following adjustments are applied to ensure that a point can be accepted from among all evaluated points during the execution of `CLS`. These adjustments also assist the Monte Carlo procedure when no reduction in the function value is observed, by allowing the Metropolis

acceptance of either a robust or a non-robust point, thereby preventing the algorithm from getting stuck.

Let dlist $= \{ \mathbf{df}_i \}$ denote the list of observed or predicted function differences and alist $= \{ \alpha_i \}$ the corresponding step sizes computed by `CLS`. The goal is to compute a new step size α_{new} and the updated function value $\mathbf{f}_{\text{new}}$ based on the information contained in dlist and alist. Let

$$\mathbf{df}_{\text{best}} = \min_i \mathbf{df}_i, \qquad i_{\text{rob}} = \operatorname*{argmin}_i \mathbf{df}_i.$$

If $\mathbf{df}_{\text{best}} < 0$, the robust step and function values are chosen by

$$\alpha_{\text{new}} = \text{alist}_{i_{\text{rob}}}, \qquad \mathbf{f}_{\text{new}} = \mathbf{f}_k + \mathbf{df}_{\text{best}}. \tag{6}$$

In this case, `CLS` finds a robust step size with a reduction in the function value. Otherwise, $\mathbf{df}_{\text{best}} \geq 0$. In this case, `CLS` fails to find such a robust step size. Instead, it identifies the point with the smallest robust change by defining the subset

$$\mathcal{I}_+ = \{ i \mid 0 < \mathbf{df}_i < \infty \}, \quad \mathbf{df}_{\text{new}} = \min_{i \in \mathcal{I}_+} \mathbf{df}_i, \qquad i_{\text{rob}} = \operatorname*{argmin}_{i \in \mathcal{I}_+} \mathbf{df}_i,$$

provided that $\mathcal{I}_+ \neq \emptyset$. Then, one of the following cases applies:
(i) If $\mathbf{df}_{\text{new}}$ exists and satisfies $\mathbf{df}_{\text{new}} \leq \Delta \mathbf{f}_k$, no further modification is applied; the robust step and corresponding function value are computed by (6).
(ii) If $\mathbf{df}_{\text{new}}$ is undefined or $\min_i \mathbf{df}_i \leq 0$, the function is almost flat:
(ii-a) The step corresponding to the largest finite element of dlist, if available, is accepted by computing

$$\mathbf{df}_{\text{new}} = \max_{i:\mathbf{df}_i < \infty} \mathbf{df}_i, \qquad i_{\text{rob}} = \operatorname*{argmax}_{i:\mathbf{df}_i < \infty} \mathbf{df}_i$$

and then substituting these two values into (6).
(ii-b) Otherwise, the largest step size is accepted by choosing

$$i_{\text{rob}} = \operatorname*{argmax}_{i:\mathbf{df}_i < \infty} \text{alist}_i$$

and substituting it into (6).
(iii) Otherwise, either a point with non-robust change or the largest almost flat step is accepted according to whether or not the condition $\mathbf{df}_{\text{new}} \leq \Delta_r \cdot \Delta \mathbf{f}_k$ holds. Here, $\Delta_r > 0$ is a tuning parameter. Indeed, if this condition holds, the robust step and corresponding function value are chosen as in (ii-b) without any further computation. Otherwise, the index i_{rob} is chosen as the largest α_i such that $\mathbf{df}_i \leq \Delta \mathbf{f}_k$. Then, the robust step and corresponding function value are computed by (6).

This formulation ensures that even under finite-precision arithmetic, where the theoretical SDC may not be numerically satisfied, a valid and stable candidate point is accepted from all evaluated points during the execution of `CLS`.

S3.2 Python interface for custom local search algorithms in `Vina`

In order to develop our custom local search algorithm for pose sampling, we developed a custom interface that allows an arbitrary Python function to be used as the local pose optimizer in `Vina`. The code is available at `https://github.com/leotaku/VinaCLS`.

A custom local optimizer is enabled by running the modified `Vina` with the command line argument `"--local_search_import"`. The value of `"--local_search_import"` should then represent the import path for a module containing the custom local optimizer and the function name in the format `path.to.module:function`.

The function pointed to by `"--local_search_import"` should have the following signature: `optimizer(fun, x_orig: conf, max_steps: int) -> (conf, float)`. Additional parameters are permissible as long as they have default variables and additional tuple elements can be used to return metadata.

The `fun` argument represents the `Vina` scoring function and provides the following methods: `fun.f(x: conf) -> float` to get the value of the scoring function for the given conformer; `fun.g(x: conf) -> array[float]` to get the gradient of the scoring function for the given conformer; and `fun.fg(x: conf) -> (float, array[float])` to retrieve the function value and gradient at the same time.

The `conf` object represents a small molecule conformer. It provides the following methods: `conf.increment(inc: array[float]) -> conf` to increment the parameters of the conformer with values from a `NumPy` array; `conf.copy() -> conf` to copy the conformer; and `len(conf) -> int`, which can be used to find the amount of parameters of the conformer.

The elements of the incrementing array are laid out as follows: The first 3 elements represent a `x, y, z` transformation of the conformer in 3D space; the following three elements represent a 3D rotation in axis-angle representation, with the norm of the three-vector describing the angle of the rotation in radians; all remaining elements represent 2D rotations around the conformers rotatable bonds, given in radians. It is important to remember that 3D rotations are not generally commutative, which often necessitates special handling in custom optimizer implementations.

To enable detailed traces, which are useful for debugging and benchmarking custom optimizers, the environment variable `"VINA_TRACES"` can be set to a writable filesystem path. Traces be written to a file at this path as lines containing tab separated values. The function `trace(type: str, event: str, value: object)` can be imported from the `vina_support` module to log events from Python.

In addition to the existing `"--max_evals"` argument, the command line arguments `"--max_gradients"` and `"--max_seconds"` can be used with our implementation to configure a maximum number of gradient evaluations and time budget for the main optimization loop. If a combination of these arguments are given, optimization will proceed until all of them are reached. If none are given, we fall back to the heuristic maximum number of steps defined by the original `Vina` implementation. Additionally, the `"--initial_pos_seed"` argument has been defined to allow seeding the initial pose randomization independently of the optimization procedure.

Figure S1 shows a simplistic example local optimizer that moves the conformer in 3D space along the negative gradient of the scoring function without adjusting its rotation or torsional angles. It also logs the best found value of the scoring function.

```python
from vina_support import conf, trace
import numpy as np

def toy_optimizer(fun, x_orig: conf, max_evals: int):
    # randomly translate ligand
    position_change = np.zeros(len(x_orig))

    # first three entries (x,y,z) are translational components
    # following four entries (x,y,z,w) are rotational components
    # remaining entries are torsional angles

    best_x, best_f = x_orig, fun.f(x_orig)

    for i in range(max_evals):
        position_change[:3] = (
           fun.g(best_x)[:3] * (-np.random.rand())
        )

        x = best_x.increment(position_change)
        if (f := fun.f(x)) < best_f:
            best_x, best_f = x, f

    trace("event", "score", best_f)
    return best_x, best_f
```

Figure S1: Example code for a custom local optimizer.

S4 Consistency of `Vina-CUDA` with `Vina`

Apart from the algorithm present in the original software, another improved line search algorithm for the `Vina` software has been published. Namely, the `RILC-BFGS` algorithm has been introduced as part of the `Vina-GPU` paper.

As such, we were interested in benchmarking our improvements against the `RILC-BFGS` algorithm as implemented in the `Vina-CUDA` or `Vina-GPU` docking programs. However, several factors result in such a comparison being inherently unreliable.

First, getting any results for a reasonable subset of our docking problems required tuning various compile-time constants related to the maximum allowed number of atoms, torsion angles and interaction pairs. While these were trivial changes, they necessarily resulted in a modifed version of the software being benchmarked, rather than the original.

Similarly, the authors of `Vina-GPU` and `Vina-CUDA` benchmarked their methods using a much different mode of operation than base `Vina`. Concretely, to make use of the high

amount of parallelism available on the GPU they ran a large number of Markov chains (8000 compared to the Vina default of 8) which a much decreased search depth. Decreasing the number of parallel chains and increasing their search depth again requires significant source-code changes, and presents the algorithm with optimization problems that were not in the scope of the original paper.

Furthermore, we also performed an investigation into the scoring function used by Vina-CUDA. We evaluated the scoring functions of Vina and Vina-CUDA at identical points, namely randomized initial ligand conformations, the native poses provided by LEADS-PEP and a selection of final poses docked by Vina and Vina-CUDA respectively. As illustrated in Figure S2 we found that for randomized poses, large differences in scoring function value were common, while large differences in gradients were common for native and best-docked poses. For randomized poses, large differences in gradients were also possible. This strongly suggests the scoring function in Vina-CUDA represents a different optimization landscape than the scoring function of Vina.

These differences are not inherently problematic when evaluating Vina-CUDA as an independent docking tool based on Vina, but necessarily make a valid score-based comparison, which is the focus of our paper, impossible.

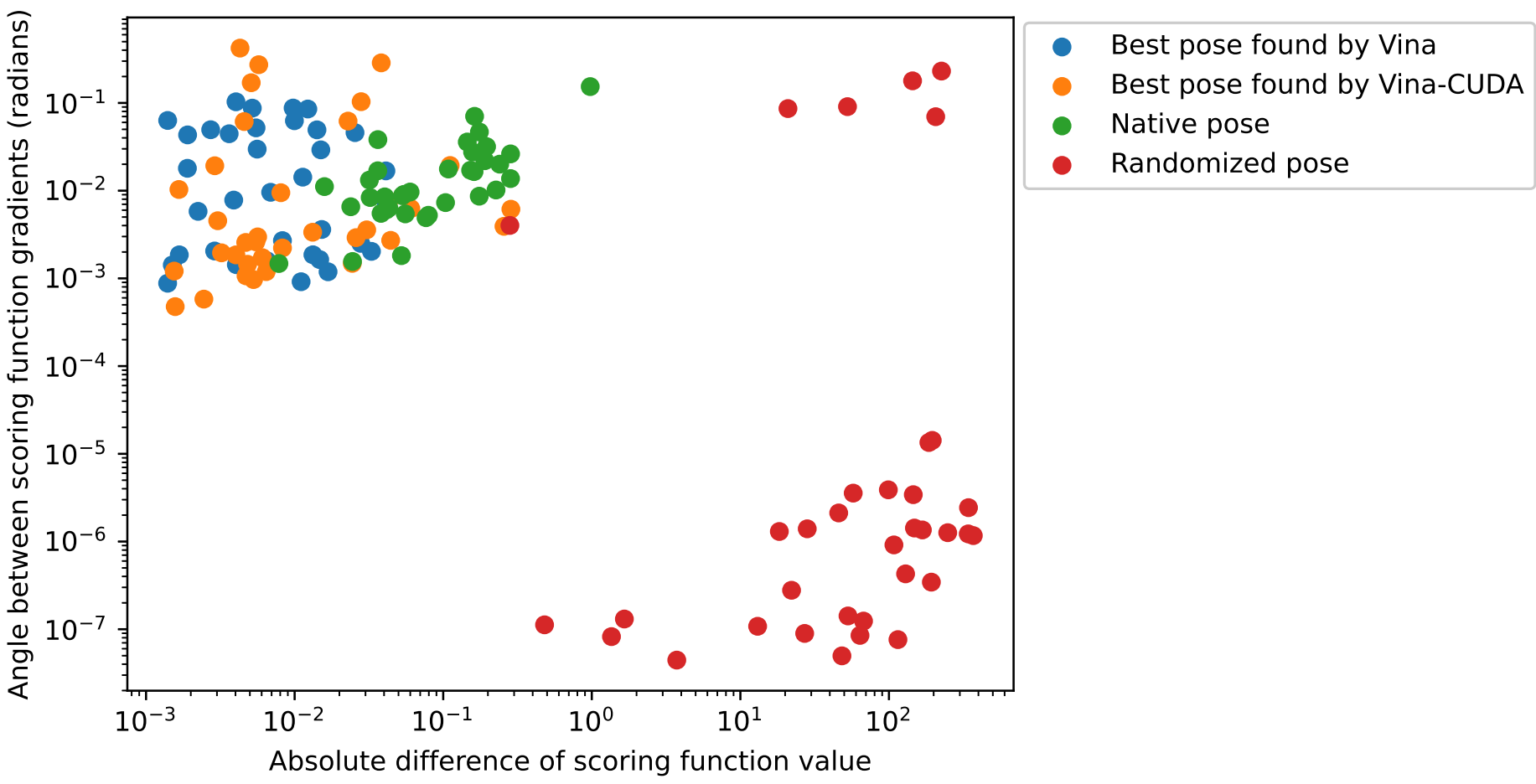

Figure S2: Differences of scoring function values and gradients between Vina and Vina-CUDA.

Given these factors, we have chosen not to include a comparison with Vina-CUDA as to not provide potentially misleading conclusions.

S5 Distribution of line search steps until acceptance

To assess the behavior of the mean number of line search iterations per BFGS iteration in different regimes (clash vs. non-clash regions), we collected initial score-step size iteration pairs for BFGS iterations from an exemplary problem (PDBBind entry 10GS, initial pose seed 1, search procedure seed 1) generated as part of our realistic PDBBind evaluation.

We filtered the obtained initial score-step size iteration pairs by initial score such that only values below a moving score threshold are retained. Figure S3 shows the mean number of attempted steps for moving score thresholds. It can be seen that `Vina` accepts steps earlier, particularly for line searches that started at well-scoring points (in this case, below -5 kcal/mol), while `Vina_CLS` was more consistent regarding the number of steps until acceptance. Also for line searches that started at particularly badly scoring points (in this case, above 100 kcal/mol), `Vina_CLS` accepted more quickly on average than `Vina`.

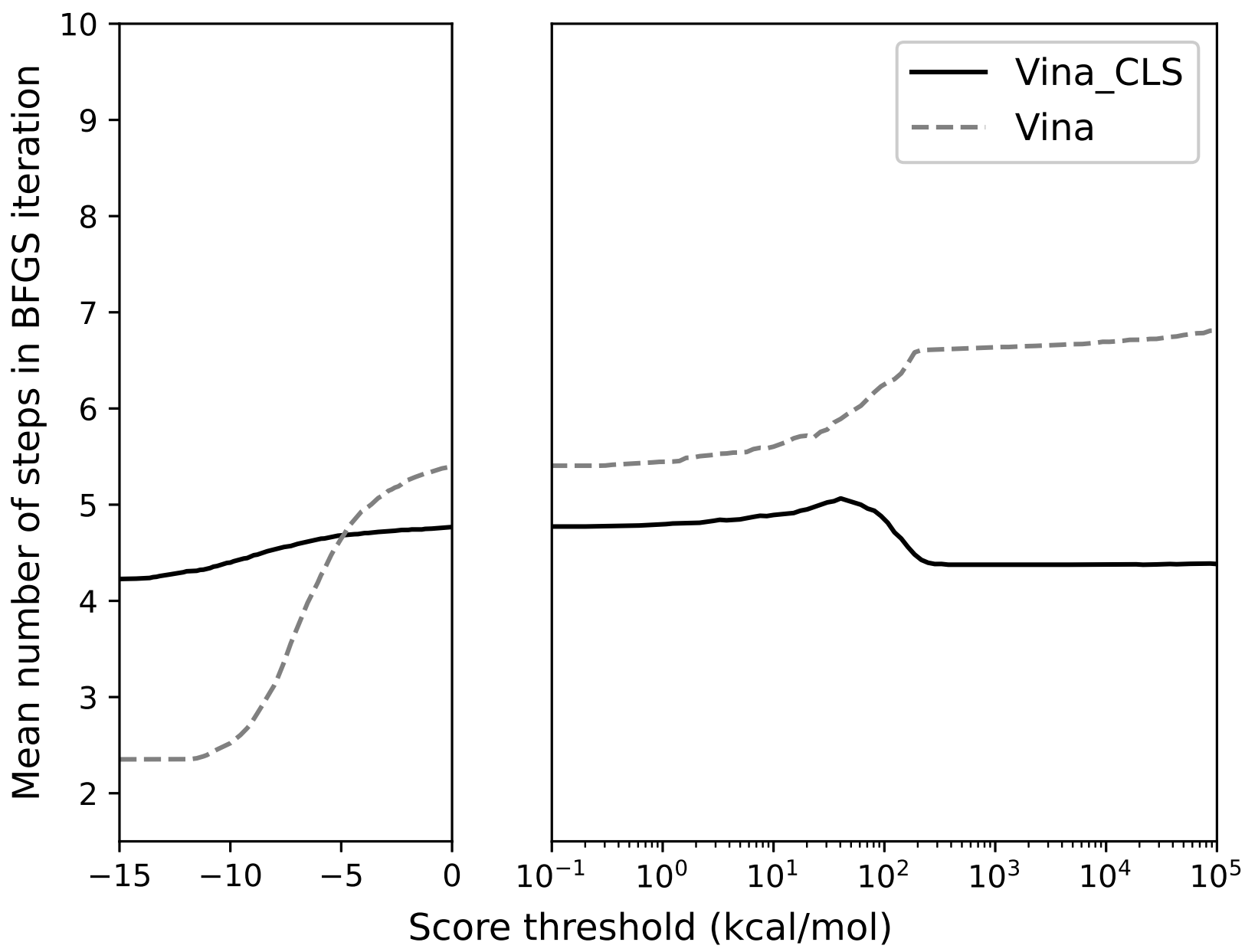

Figure S3: Mean number of steps in BFGS iterations which start below a score threshold.

Given `Vina` accepted steps earlier in well-scoring regions while `Vina_CLS` accepted trial points more quickly in regions with score above 100 kcal/mol, we provide two separate histograms for the number-of-steps distribution of all problems in the redocking experiment. In Figure S4 we show both the distributions for number of step sizes of BFGS iterations starting at points with scoring function values above (noninformative) and below 0 kcal/mol (informative). When starting from an informative point, the `Vina_CLS` algorithm performed a larger number of iterations on average, while when starting from a noninformative point, the algorithm prefered smaller numbers of iterations and hit the cap on the maximum number of iterations much more rarely.

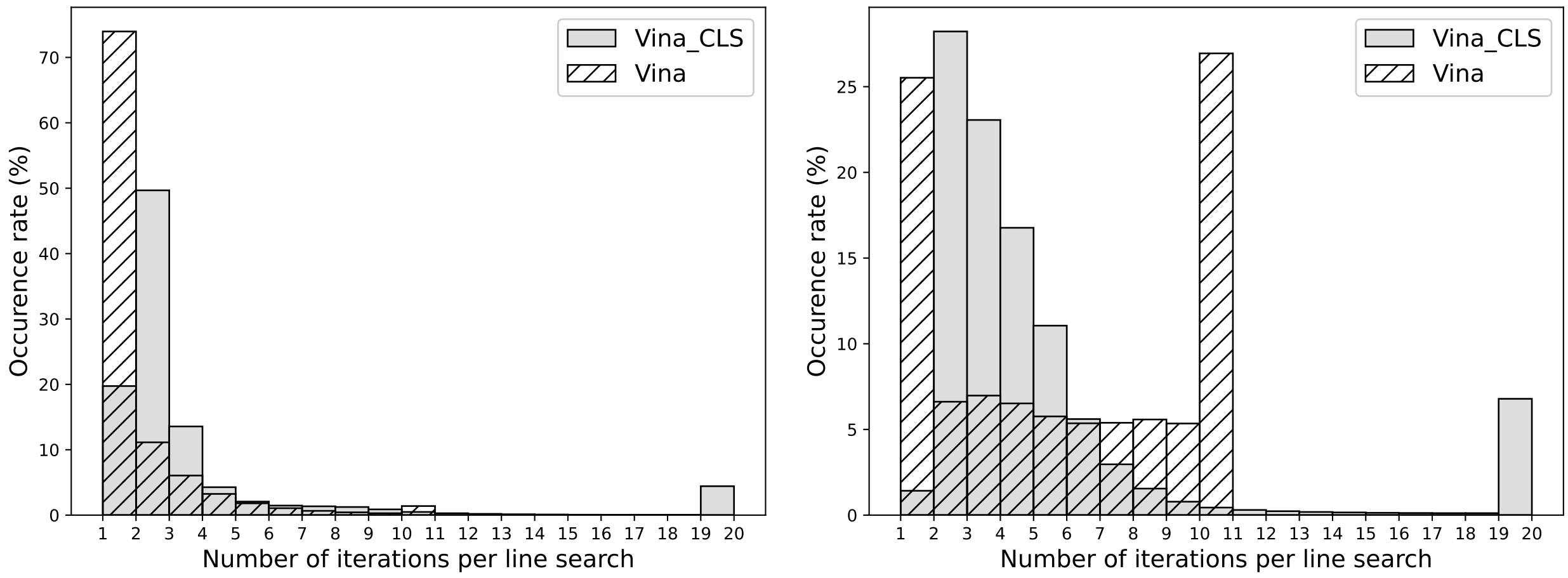

(a) Score ≤ 0 kcal/mol (b) Score > 0 kcal/mol

Figure S4: Histograms of number of attempted steps for line searches starting at points scored below (a) and above (b) 0 kcal/mol. Note, that for `Vina_CLS` always at least two steps are attempted due to fast convergence regions that are potentially almost quadratic. For these plots, we consider the `Vina_CLS` algorithm to terminate in one step if the first step attempted step is accepted over the second step.

S6 Stratified analysis of pose evaluation

To assess whether ligand or protein information has a major impact the result that `Vina_CLS` exhibits heightened redocking performance across `RMSD` thresholds, we partitioned the protein-ligand pairs into approximately equally sized bins according to the corresponding chemical or biological information and calculated the top-1 $\Delta(t)$ relative to the bin size. For this stratified analysis of redocking performance, we reused the data (4850 prepared protein-ligand pairs from `PDBbind` refined set) from the pose evaluation section in the main document.

To assess whether ligand chemistry changes the heightened redocking performance of our algorithm compared to the baseline, we first considered ligand descriptors that are correlated with molecular flexibility. Namely, Figure S5 shows results stratified by the number of rotatable bonds in the ligand as well as the number of heavy atoms. It can be seen that for ligands with fewer than 3 rotatable bonds the improvement in redocking performance provided by `Vina_CLS` was consistently lower than for all other ligands. Number of heavy atoms also positively correlated with improvement in redocking performance using `Vina_CLS`, except for the bin containing ligands with the most heavy atoms, which, unintuitively, show decreased improvement in the relevant region compared to the next bin.

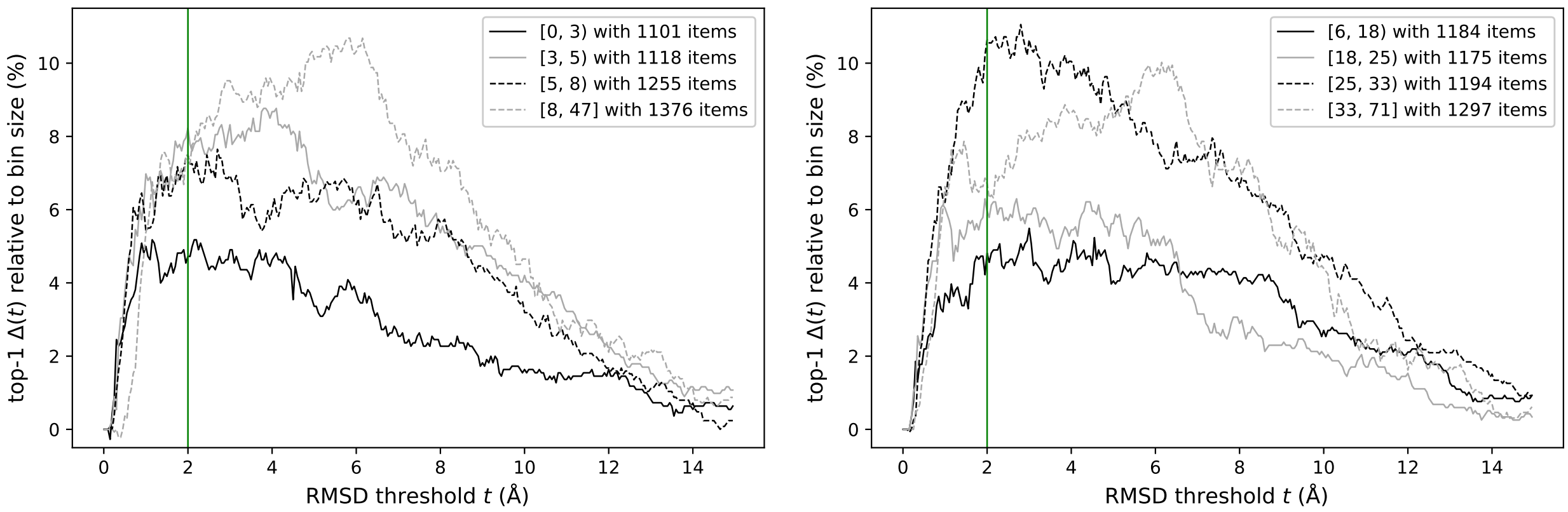

(a) Stratified by number of rotatable bonds (b) Stratified by number of heavy atoms

Figure S5: Additional poses found by Vina CLS compared to Vina given various RMSD thresholds among top-1 scored poses, stratified by number of rotatable bonds (a) and number of heavy atoms (b). The conventional threshold of 2 Å is marked in green.

To assess whether the geometry of the protein pockets changes the heightened redocking performance of our algorithm compared to the baseline, we considered protein pocket descriptors generated using `fpocket` [7] in `dpocket` mode. For this analysis we excluded 36 entries in our dataset where `fpocket` failed to find a suitable pocket. Following the `fpocket` user manual, we used the number of alpha spheres inside the pocket as an approximation of the pocket size. We also used the mean alpha sphere radius as an approximation of the local tightness of the pocket. Visual inspection of Figure S6 suggests some correlation between pocket tightness and improved redocking performance when using `Vina_CLS`.

However, given the modest difference in performance across all of shown stratification strategies, as well as the impact of stochasticity due to the Monte Carlo global search, we suggest that these results should be interpreted with caution.

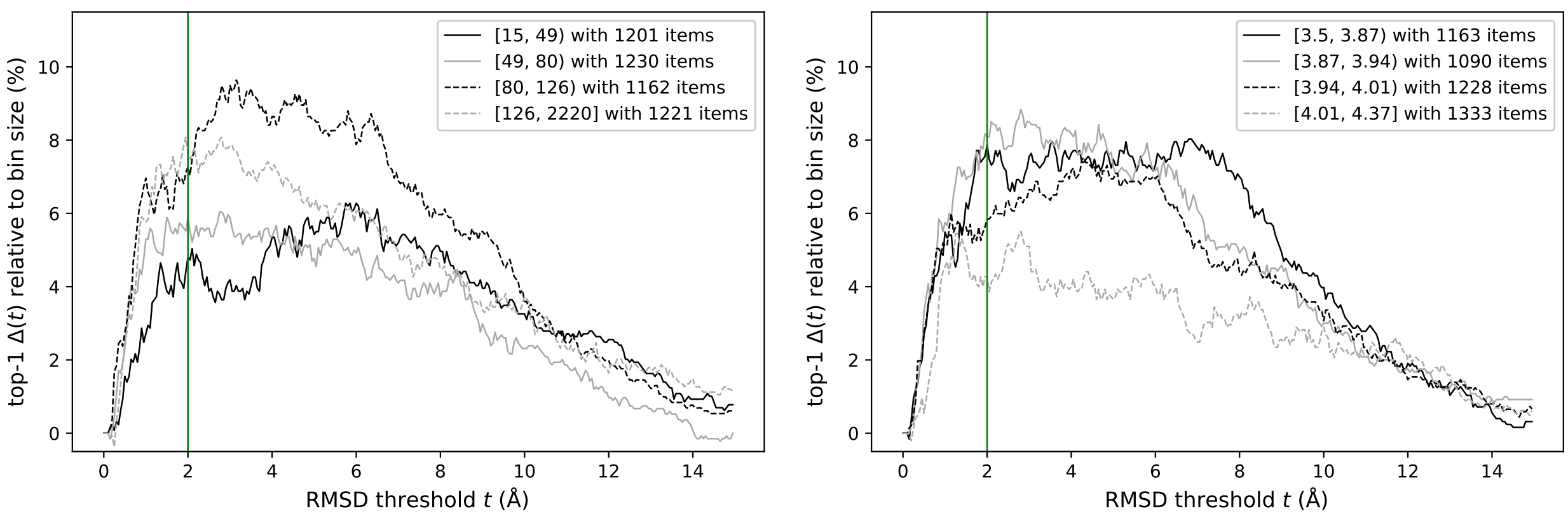

(a) Stratified by number of alpha spheres (b) Stratified by mean radius of alpha sphere

Figure S6: Additional poses found by Vina CLS compared to Vina given various RMSD thresholds among top-1 scored poses, stratified by number `fpocket`-generated alpha spheres (a) and mean alpha sphere radius (b). The conventional threshold of 2 Å is marked in green.

References

[1] Eberhardt, J.; Santos-Martins, D.; Tillack, A. F.; Forli, S. AutoDock Vina 1.2.0: New Docking Methods, Expanded Force Field, and Python Bindings. *J. Chem. Inf. Model.* **2021**, *61*, 3891–3898.

[2] Trott, O.; Olson, A. J. AutoDock Vina: Improving the speed and accuracy of docking with a new scoring function, efficient optimization, and multithreading. *J. Comput. Chem.* **2009**, *31*, 455–461.

[3] Liu, D. C.; Nocedal, J. On the limited memory BFGS method for large scale optimization. *Math. Program.* **1989**, *45*, 503–528.

[4] Neumaier, A.; Kimiaei, M.; Azmi, B. Globally linearly convergent nonlinear conjugate gradients without Wolfe line search. *Numerical Algorithms* **2024**, *97*, 1607–1633.

[5] Neumaier, A.; Kimiaei, M. An improvement of the Goldstein line search. *Optim. Lett.* **2024**, *18*, 1313–1333.

[6] Kimiaei, M.; Neumaier, A.; Azmi, B. LMBOPT: a limited memory method for bound-constrained optimization. *Math. Program. Comput.* **2022**, *14*, 271–318.

[7] Le Guilloux, V.; Schmidtke, P.; Tuffery, P. Fpocket: An open source platform for ligand pocket detection. *BMC Bioinform.* **2009**, *10*.
